# Feed‐backs among inbreeding, inbreeding depression in sperm traits, and sperm competition can drive evolution of costly polyandry

**DOI:** 10.1111/evo.13363

**Published:** 2017-11-13

**Authors:** Greta Bocedi, Jane M. Reid

**Affiliations:** ^1^ School of Biological Sciences University of Aberdeen Zoology Building Tillydrone Avenue Aberdeen AB24 2TZ United Kingdom

**Keywords:** Effective population size, female multiple mating, fertility assurance, inbreeding, population structure, population‐wide homozygosity, sperm competition

## Abstract

Ongoing ambitions are to understand the evolution of costly polyandry and its consequences for species ecology and evolution. Emerging patterns could stem from feed‐back dynamics between the evolving mating system and its genetic environment, defined by interactions among kin including inbreeding. However, such feed‐backs are rarely considered in nonselfing systems. We use a genetically explicit model to demonstrate a mechanism by which inbreeding depression can select for polyandry to mitigate the negative consequences of mating with inbred males, rather than to avoid inbreeding, and to elucidate underlying feed‐backs. Specifically, given inbreeding depression in sperm traits, costly polyandry evolved to ensure female fertility, without requiring explicit inbreeding avoidance. Resulting sperm competition caused evolution of sperm traits and further mitigated the negative effect of inbreeding depression on female fertility. The evolving mating system fed back to decrease population‐wide homozygosity, and hence inbreeding. However, the net overall decrease was small due to compound effects on the variances in sex‐specific reproductive success and paternity skew. Purging of deleterious mutations did not eliminate inbreeding depression in sperm traits or hence selection for polyandry. Overall, our model illustrates that polyandry evolution, both directly and through sperm competition, might facilitate evolutionary rescue for populations experiencing sudden increases in inbreeding.

Understanding key forces that drive the evolution and persistence of complex mating systems remains a central endeavor in evolutionary biology. One overarching hypothesis is that mating systems will be substantively influenced by interactions among kin, encompassing inbreeding alongside kin competition and cooperation (Charlesworth and Charlesworth 1987; Hatchwell 2009; Szulkin et al. 2013; Shuker and Simmons 2014). Mating system evolution must then depend on aspects of life‐history variation and population ecology that shape the frequencies of different types of relatives that coexist within any population (hereafter “population relatedness structure”), and hence shape the opportunities for reproductive interactions among different relatives (e.g., Banks et al. 2005; Hatchwell 2009; Beckerman et al. 2011; Szulkin et al. 2013; Pizzari et al. 2015). In turn, by altering social structures and distributions of reproductive success, evolving mating systems can themselves feed back to further shape population relatedness structure and population ecology, and hence shape genetic and genotypic variation and inbreeding load (Pamilo 1985; Ross 2001; Lehtonen and Kokko 2012; Holman and Kokko 2013; Pizzari and Wedell 2013; Taylor et al. 2014; Abu Awad and Billiard 2017). The potential for such feed‐backs is well established for self‐fertilization and associated evolution of inbreeding depression (e.g., Gervais et al. 2014; Kamran‐Disfani and Agrawal 2014; Porcher and Lande 2016; Abu Awad and Billiard 2017). However, very little theory has explicitly considered the evolutionary dynamics of mating systems in the context of dynamic distributions of relatedness arising in nonselfing systems, or thereby fully captured evolutionary feed‐backs between mating systems, population relatedness structure, and genetic variation (Duthie and Reid 2016; Duthie et al. 2016).

One prime example concerns the ongoing challenge of explaining the evolution of polyandry, defined as female mating with multiple males within a single reproductive bout (Parker and Birkhead 2013; Pizzari and Wedell 2013), which is especially problematic when such polyandry imposes direct costs on females (Rowe 1994; Wigby and Chapman 2005; Sardell et al. 2012; Slatyer et al. 2012). Polyandry is widely hypothesized and observed to evolve in the context of inbreeding (Stockley et al. 1993; Jennions and Petrie 2000; Simmons 2005; Michalczyk et al. 2011), and hence as a property of populations where relatives interact. Such polyandry might evolve because of indirect selection arising if multiple mating facilitates inbreeding avoidance through pre‐ and/or postcopulatory mate choice; females could use either route to allocate paternity to a less closely related male and hence reduce inbreeding depression in offspring viability (Jennions and Petrie 2000; Tregenza and Wedell 2002; Duthie et al. 2016). However, there are multiple mechanisms by which polyandry evolution could be directly affected by, and directly affect, inbreeding without requiring evolution of complex mechanisms of inbreeding avoidance enacted through mate choice and underlying kin discrimination. Specifically, inbreeding might directly affect aspects of male or female reproductive biology, such as fertility (e.g., Saccheri et al. 2005; Fox et al. 2012; Losdat et al. 2014), and thereby cause direct selection on polyandry. Emerging polyandry might then alter the variance in both sexes’ reproductive success and the form of reproductive skew, and thereby alter effective population size and hence the mean degree of inbreeding (Webster et al. 1995; Holman and Kokko 2013; Taylor et al. 2014). Further, polyandry can generate half‐sibs rather than full‐sibs, and hence other half‐relatives, and thereby directly affect population relatedness structure and individuals’ risks of close inbreeding (Cornell and Tregenza 2007).

Some theoretical models have examined single components of evolutionary feed‐backs that could potentially arise between polyandry and inbreeding, for example, concerning effects of polyandry on effective population size (Lotterhos 2011) or sibship structures (Cornell and Tregenza 2007). However, while much attention has been devoted to investigating if and how polyandry could evolve due to indirect selection stemming from inbreeding avoidance (Jennions and Petrie 2000; Tregenza and Wedell 2002; Michalczyk et al. 2011; Duthie et al. 2016), the possibility that polyandry could evolve due to direct selection stemming from effects of inbreeding on reproducing individuals has not been similarly explicitly considered, either in isolation or in combination with internally consistent feed‐backs. Consequently, we lack theory that fully addresses how polyandry evolution can be directly driven by inbreeding, and conversely how population relatedness structure and inbreeding risk are affected by evolving polyandry, and hence that encompasses dynamic evolutionary feed‐backs between costly mating systems and their ecological and genetic environment.

Here, we lay out a mechanism by which inbreeding could cause evolution of costly polyandry and thereby generate feed‐backs that affect the degree and consequences of inbreeding (Fig. 1). Specifically, in populations where inbreeding occurs, direct selection for polyandry could arise if there were inbreeding depression in key gametic traits expressed by inbred males (e.g., sperm or ejaculate traits), and hence inbreeding depression in male fertility. Polyandry might then evolve as insurance against female infertility when females risk mating with inbred males, even given direct costs of multiple mating (Fig. 1A–D). Polyandry would thus evolve as means to avoid the direct negative consequences of females mating with inbred males, as opposed to the indirect negative consequences of inbreeding with related males (e.g., Tregenza and Wedell 2002; Cornell and Tregenza 2007; Duthie et al. 2016). Multiple ecological and evolutionarily feed‐backs might then ensue (Fig. 1), which might change the levels of inbreeding and inbreeding depression in the population, and hence affect selection on polyandry.

**Figure 1:**
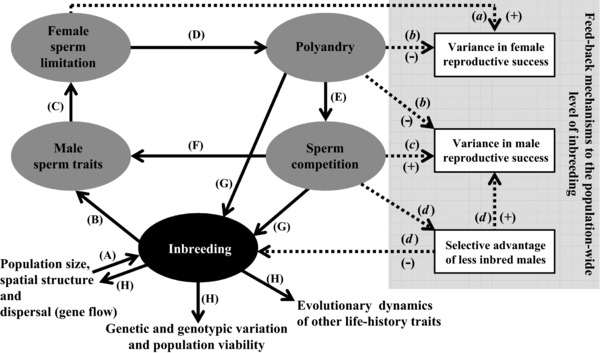
Conceptual representation of potential evolutionary feed‐backs between inbreeding, sperm traits, polyandry, and sperm competition. Inbreeding occurring in small or viscous populations (A) depresses sperm traits (B), increasing risk of fertilization failure due to female sperm limitation (C). This imposes direct selection on females to mate multiply (i.e., polyandry) to ensure fertility (D). Resulting polyandry creates sperm competition (E), driving further evolution of sperm traits (F), which may feed back to affect female sperm limitation and hence polyandry. The evolved mating system may then feed back to alter the population‐wide level of inbreeding (G), potentially affecting genetic and genotypic variation, population viability, and life‐history evolution (H). The form of this feed‐back (G) is not straightforward to predict because polyandry, sperm competition, and inbreeding depression could have multiple congruent or conflicting effects on the mean and variance in both sexes’ reproductive success (dashed arrows, *a*–*d*; plus and minus symbols denote hypothesized increases and decreases, respectively).

First, the occurrence of inbreeding depression in male sperm traits, and consequent polyandry and resulting sperm competition, might drive further coevolutionary dynamics of sperm traits and the overall mating system (Fig. 1B–G). In general, inbreeding depression in male mating traits and performance can be exacerbated by sexual selection and male–male competition (Meagher et al. 2000; Joron and Brakefield 2003; Agrawal 2011; Janicke et al. 2013). The magnitude of inbreeding depression in sperm traits, or in a male's general ability to allocate resources to competing sperm or ejaculate traits, could therefore be affected by the intense sperm competition that is itself caused by initial polyandry. Indeed, inbreeding can substantially reduce a male's sperm competitive ability (Zajitschek et al. 2009; Michalczyk et al. 2010; Simmons 2011), and sperm competition can drive evolution of male sperm traits (Parker and Pizzari 2010; Fig. 1F) and of polyandry itself (Fig. 1C–D; Engqvist 2012; Alonzo and Pizzari 2013; Abe and Kamimura 2015; Bocedi and Reid 2016).

Second, such dynamics of polyandry and resulting sperm competition might in turn affect the variance in male and female reproductive success and hence effective population size, with further consequences for population‐wide inbreeding and resulting homozygosity (Fig. 1a–d). However, the net effect of evolving polyandry on the variance in both sexes’ reproductive success is hard to predict (Holman and Kokko 2013). Polyandry evolving under direct selection for fertility assurance might decrease the variance in female reproductive success by reducing female infertility (Fig. 1b), hence increasing effective population size. Meanwhile, polyandry might increase the variance in male reproductive success (Fig. 1c) because it increases male–male competition for mating and creates sperm competition, and thereby skews the distribution of paternity arising if males differ in mating success and competitive fertilization ability (Webster et al. 2007; Lotterhos 2011). Conversely, if males are relatively similar in their mating and fertilization probability, increased polyandry might lead to more evenly shared paternity, thereby decreasing the variance in male reproductive success (Fig. 1B; Balloux and Lehmann 2003; Pearse and Anderson 2009; Lotterhos 2011). Indeed, increased female mating frequency can decrease the average paternity share of males that would otherwise be relatively successful, causing investment in sperm traits to decrease (Parker and Pizzari 2010).

Third, given inbreeding depression in male gametic traits, the occurrence of intense sperm competition caused by polyandry could generate a strong negative association between a male's own level of inbreeding and his reproductive success, skewing paternity toward less inbred males (Fig. 1d). If homozygosity is heritable, as can occur (e.g., Mitton et al. 1993; Reid et al. 2006; Nietlisbach et al. 2016), resulting offspring might be less homozygous on average than otherwise expected. The specific form of reproductive skew generated by the mating system might thereby cause lower population‐wide homozygosity than otherwise expected given the variance in reproductive success.

The relative importance of, and interactions among, these mechanisms will act alongside direct effects of polyandry on sibship structures (Cornell and Tregenza 2007) to determine the net effect of the evolving polyandrous mating system on individual and population‐wide inbreeding and resulting homozygosity. Such changes could then potentially alter selection on mechanisms of inbreeding avoidance including mate choice and dispersal, thereby affecting the eco‐evolutionary dynamics of inbreeding itself, and also affecting population viability and evolution of other life‐history traits (Fig. 1H).

The general hypothesis that polyandry evolution could be driven by fertility assurance has been widely proposed in contexts other than inbreeding (Sheldon 1994; Birkhead and Pizzari 2002; Hasson and Stone 2009; Forbes 2014), but remains somewhat contentious because of the expectation that male infertility might be rapidly eliminated due to strong direct selection (Arnqvist and Kirkpatrick 2005; Griffith 2007; Forstmeier et al. 2014). However, the potentially diverse causes of male infertility, coupled with evolutionary sexual conflict, have been suggested to maintain some degree of infertility (Hasson and Stone 2009). Indeed, there is good evidence that male infertility is maintained in natural populations, with variable prevalence (García‐González 2004; Rhainds 2010; Tyler and Tregenza 2013; Greenway and Shuker 2015; Greenway et al. 2015). There is also empirical evidence that inbreeding can affect numerous male gametic traits across diverse animal and plant systems (Losdat et al. 2014), concurring with the general expectation that inbreeding depression in fitness‐related traits will be strong (DeRose and Roff 1999). For example, in seed beetles *Callosobruchus maculatus* inbred males’ ejaculates contained fewer sperm, meaning that females that mated with inbred males had reduced fertility (Fox et al. 2012). However, despite such established background, the hypothesis that inbreeding could drive evolution of polyandry through inbreeding depression in male gametic traits, and thereby drive feed‐backs that affect inbreeding (Fig. 1), has not been explicitly considered.

We built a genetically explicit individual‐based model to test the hypothesis that costly polyandry can evolve as a female strategy to reduce the fitness costs of mating with inbred males (rather than the fitness costs of inbreeding with relatives), and to quantify resulting feed‐back dynamics among polyandry, sperm competition, and population‐wide inbreeding (Fig. 1). Taking a genetically explicit individual‐based approach allows population relatedness structure, and hence the degrees of individual and population‐wide inbreeding, to emerge from evolving polyandry and sperm traits alongside population spatial structure stemming from restricted dispersal. Our model thereby considers mating system evolution in the context of an internally consistent ecological and genetic environment, and allows full consideration of the complex ecological and evolutionary feed‐backs, and mechanisms underlying them, that might arise. Furthermore, genetically explicit modeling of inbreeding depression allows purging, and hence evolution of inbreeding depression itself, to be considered.

First, we test the hypothesis that inbreeding depression in sperm traits can cause evolution of polyandry by exacerbating female sperm limitation and hence infertility risk, even when polyandry imposes a direct cost on females (Fig. 1B–D). Second, we determine how, and the degree to which, sperm competition resulting from initial polyandry can feed back to affect further evolution of sperm traits and polyandry (Fig. 1E–F). We thereby examine the relative contributions of co‐occurring inbreeding depression in sperm traits and emerging sperm competition in driving female sperm limitation and polyandry. Third, we determine to what extent the evolving mating system feeds back to affect the population‐wide degree of inbreeding and consequent homozygosity (Fig. 1G), and determine the underlying mechanisms (Fig. 1a–d). Finally, we examine the effect of the evolving mating system on female fertility, and consider the implications for population fitness (Fig. 1H).

## The Model

We embedded components of a model designed to represent coevolutionary dynamics of polyandry and sperm traits (Bocedi and Reid 2016) into a spatially explicit framework for modeling population relatedness structure, inbreeding, and inbreeding depression. We modeled a system of 25 subpopulations of a dioecious species, structured on a 5 × 5 grid and connected by dispersal. This spatial structure allows relatedness structure and the degree of inbreeding within each subpopulation, and across the entire population, to emerge from the modeled dynamics. Specifically, we vary the level of inbreeding occurring within subpopulations among different simulations by varying dispersal probability, thereby creating different degrees of population genetic structure. By allowing the level of inbreeding to emerge from the population structure, rather than imposing a fixed inbreeding coefficient, we captured effects of the evolving mating system on the level of inbreeding, and thus examined potential feed‐backs (e.g., Fig. 1). Generations are nonoverlapping. At each generation, individuals experience, in sequence, reproduction (mating, fertilization, and offspring birth), adult mortality, offspring dispersal, and density‐dependent survival. All model variables and parameters are summarized in Table S1.

### GENETIC ARCHITECTURE

We model three evolving traits (following Bocedi and Reid 2016); female remating interval (τ, which controls the female's mating rate and hence the degree of polyandry, 1/τ), and two male sperm traits: sperm number (*s*) and sperm mortality rate (μ). We assume a diploid autosomal additive genetic system, where all individuals of both sexes carry genes underlying τ, *s*, and μ. Each of the three traits is determined by 20 unlinked loci with a continuous distribution of alleles (Kimura 1965) and no pleiotropy. The initial value of each allele is sampled from a normal distribution with set initial mean and variance (Table S1). Alleles experience a mutation probability of 0.001/allele/generation; when mutation occurs a random normal deviate with mean zero is added to the allele value (Table S1). Each individual's genotypic value for τ, *s* and μ (hereafter *g*_τ_, *g_s_*, and *g_μ_*) is the sum of its 40 associated allelic values. Phenotypic expression is sex limited with no environmental variance. We assume 0.01 ≤ τ ≤ 1, so that females always mate once, but no more than 100 times, per egg to be fertilized; *s* ≥ 1 so that males cannot produce less than one sperm cell; and μ ≥ 10^−10^ to avoid numerical errors given by μ = 0.

We implement a genetically explicit model for inbreeding depression (e.g., Guillaume and Perrin 2006; Duthie and Reid 2016). Individuals have 1000 unlinked autosomal loci carrying deleterious mutations. Alleles at each locus can assume either a value of 1 (“wild type” allele with no deleterious effect), or 0 (deleterious mutation). At the beginning of each simulation, all individuals are initialized with no deleterious mutations. Alleles then mutate with probability 0.001/allele/generation. Mutations are bidirectional, meaning that wild‐type alleles can mutate to deleterious alleles and vice versa (e.g., Roze and Rousset 2004; Duthie and Reid 2016). Deleterious mutations have an effect *S* in the homozygous state, and dominance coefficient *h* (Higgins and Lynch 2001). We assume deleterious mutations only affect males’ traits, and focus primarily on the case where mutations decrease *s* (but see Supporting Information S7). Here, a male *i*’s phenotype is given by:

(1) $$s_{i}=g_{s\;,i}{\left(1-S\right)}^{\Theta }{\left(1-hS\right)}^{\theta },$$

where Θ and θ are the number of loci that are homozygous and heterozygous for deleterious mutations, respectively. This multiplicative model assumes each mutation independently affects the phenotype (Charlesworth and Charlesworth 1987; Mills and Smouse 1994). We assume *h* = 0 for all mutations such that only loci that are homozygous for deleterious mutations contribute to inbreeding depression. Each male's *s* is therefore negatively affected by the overall genetic load, which depends on mutation rate, on the degree of inbreeding that affects the number of homozygous deleterious loci, and on *S*.

To track the degree to which individuals are inbred (i.e., are the progeny of related mates), we model an additional 1000 neutral autosomal diploid loci with continuously distributed allelic values and mutation probability of 0.001/allele/generation. Each individual allele is initialized with a value sampled from the real uniform distribution *U*[−1000.0, 1000.0] and values of mutated alleles are drawn from the same distribution. Alleles at the same locus will be identical only by descent as the chance of nondescent identity by state, stemming from initialization or mutation, is negligible. We define an individual's neutral homozygosity (H_i_) as the number of homozygous loci/1000, which represents a proxy for the realized individual coefficient of inbreeding (Markert et al. 2004; Neff and Pitcher 2008; Fromhage et al. 2009). This method allows us to determine the effect of inbreeding depression in sperm traits, and consequent evolving mating system, on the population‐wide degree of inbreeding, independently of homozygosity at the loci that cause inbreeding depression and might consequently experience purging (Supporting Information S1).

### REPRODUCTION, DISPERSAL, AND SURVIVAL

At each generation, each female sequentially produces *R* = 8 eggs that are fertilized at the discrete times *t* = 1, 2, 3… *R* (following Bocedi and Reid 2016). Females mate with randomly drawn males from within their subpopulation. Each female mates once at *t* = 0. Subsequently, females mate deterministically at the remating interval given by their phenotype τ. If τ < 1, a female mates with multiple males per egg (i.e., polyandry), whereas if τ = 1, the female mates only once per fertilization event (i.e., monandry). If 1/τ is not an integer, a female mates, on average, 1/τ times at each fertilization event, and a total number of times equal to the largest integer ≤ *R*/τ, plus the initial mating at *t* = 0.

At each mating, male *i* transfers *s_i_* sperm to the female. The number of sperm ζ*_i_* still viable at the time of fertilization *t* depends on *s_i_*, on the male's sperm mortality rate (μ*_i_*), and the time at which he mated with the female (*t_i_*; as determined by the female's remating interval) (Parker 1998; Engqvist 2012):

(2) $$\zeta_{i,t}=s_{i}e^{-\mu_{i}\left(t-t_{i}\right)}.$$

At each fertilization event, all remaining viable sperm of all the female's previous mates (*N_mates_*) compete to fertilize the egg through a “fair‐raffle” (Parker and Pizzari 2010) weighted by each male's number of viable sperm, where male *i*’s fertilization probability is:

(3) $$\phi_{i,t}=\frac{\zeta_{i}}{\sum_{j=1}^{N_{mates}}\zeta_{i}}\;.$$

This process generates internally consistent half‐sibships contingent on the degree of a female's polyandry and the competitive fertilization ability of her mates, as determined by sperm number and mortality rate.

Female sperm limitation is assumed to occur, such that the probability of egg fertilization (Φ) increases with the total viable sperm available to the female at the time of fertilization (*Ζ_t_*), which can comprise viable sperm remaining from previous fertilization events:

(4) $$\Phi_{t}=1-e^{-rZ_{t}},$$

where *r* is the probability that each sperm will fertilize the egg (Schwartz et al. 1981; Alonzo and Pizzari 2013; Bocedi and Reid 2016). Such female sperm limitation is widespread in nature. Although numerous sperm cells are produced, they suffer very high mortality, including due to female adaptations driven by sexually antagonistic selection, involving risk of oligospermy versus polyspermy and cryptic female choice (Birkhead et al. 1993; Eberhard 1996; Holland and Rice 1999), sperm competition, and trade‐offs in male allocations (Wedell et al. 2002; García‐González 2004; Hasson and Stone 2009).

Female multiple mating and male sperm traits are assumed to be costly such that high values reduce individual viability (*v*), defined as the probability that an individual survives to the end of the reproductive phase (*t* = *R*). An individual *i*’s survival probability (ψ) at time *t* is given by:

(5) $$\psi_{i,t}=\nu_{i}^{t/R}.$$

Hence, throughout the reproductive phase, individual mortality rate is –ln(ν*_i_*)/*R*. Individual mortality occurs as discrete event immediately after each fertilization event. Female *i*’s viability depends on her number of matings (*R*/τ*_i_*) and on the strength of direct selection against female multiple mating (*ω^2^_f_* = 1.28 × 10^5^; see Supporting Information S2), such that:

(6) $$\nu_{i}=e^{-{\left(R/\tau_{i}-R\right)}^{2}/2\omega_{f}^{2}}.$$

Males can invest a finite amount of resources (ρ_0_) into their sperm traits *s* and μ with no cost to their own survival, but can increase the total resources invested into sperm (ρ) at a cost. Male *i*’s viability depends on the resources *ρ_i_* invested in sperm and on the strength of direct selection against such investment ($\omega_{m}^{2}$ = 1; see Supporting Information S2):

(7) $$\nu_{i}=\left\{\begin{array}{c} e^{-{\left(\rho_{i}-\rho_{0}\right)}^{2}/2\omega_{m}^{2}}\;\;\;\;\;\rho_{i}>\rho_{0}\\ 1\;\;\;\;\;\;\;\;\;\;\;\;\;\;\;\;\;\;\;\;\;\;\;\;\;\;\;\rho_{i}\leq \rho_{0} \end{array}\right..$$

We assume the males’ two evolving sperm traits, *s* and μ, trade‐off such that the amount of resources a male *i* allocates to sperm is:

(8) $$\rho_{i}=s_{i}\beta \frac{1}{\mu_{i}},$$

where β determines the cost of a single sperm cell (Engqvist 2012; Bocedi and Reid 2016). There is ample empirical evidence that sperm is costly (Wedell et al. 2002; Pitnick et al. 2009; delBraco‐Trillo et al. 2016), and that increased investment in sperm following increased sperm competition is likely to trade‐off against other male traits (Snook 2005), including other sperm traits (Moore et al. 2004; Helfenstein et al. 2008; Evans 2011; Immler et al. 2011) as well as survival (Van Voorhies 1992). We applied a trade‐off between *s* and μ to examine the potential interactions between sperm competition, allocation to different sperm traits and inbreeding depression in driving polyandry evolution, and to facilitate comparison with previous models that did not consider inbreeding, or resulting inbreeding depression in sperm traits (Engqvist 2012; Bocedi and Reid 2016). Although results will depend quantitatively on the specific form of the trade‐off, they are likely to be qualitatively robust to alternative formulations (Supporting Information S3). Males therefore experience a three‐way trade‐off: they can trade‐off *s* against μ with no cost to their own survival, or they can increase their investment in *s* and/or μ at a cost of reduced survival. The resource a male invests into sperm (ρ) is determined by the male's phenotype prior to imposing inbreeding depression. Thus, inbred males pay the same cost as outbred males with the same genotypic value for *s* (i.e., *g_s_*), and inbreeding depression affects *s* but does not directly affect *ρ*, and hence male survival, for a given genotype.

After reproduction, all adults die and offspring (birth sex‐ratio 1:1) disperse among subpopulations with probability *d*. Dispersal distance and direction are sampled from a negative exponential distribution (mean 1.5 cells) and uniform distribution between 0 and 2π, respectively, and are resampled if the destination falls outside the grid. After dispersal, density‐dependent mortality occurs such that individuals in each subpopulation survive with probability min(*K*/*N*, 1), where *K* is the carrying capacity (set to 160 individuals per cell) and *N* is the total number of individuals.

### SIMULATION EXPERIMENTS

We first ran sets of simulations to test the initial premise that inbreeding depression in sperm number can exacerbate female sperm limitation and drive an evolutionary increase in polyandry, even when polyandry imposes a direct survival cost on females (e.g., Fig. 1B–D). We repeated simulations across increasing strengths of selection against deleterious mutations (*S* = 0, 0.002, 0.006, 0.01, 0.02, 0.03 0.04, and hence increasing strengths of inbreeding depression in *s*), and different levels of inbreeding obtained by setting three different dispersal probabilities (*d* = 0.1, 0.01, and 0.001).

Second, to elucidate how sperm competition resulting from initial polyandry feeds back to affect evolution of sperm traits and polyandry, and to tease apart the relative contributions of inbreeding depression versus sperm competition in shaping mating system evolution (Fig. 1B vs. F), we ran simulations where we excluded sperm competition. Here, females could still evolve to mate multiple times, and experienced direct costs. However, for each egg, females that mated multiply mated repeatedly with the same male rather than with different males.

Third, to determine how the evolving mating system feeds back to affect the overall level of inbreeding and resulting homozygosity, we computed population‐wide homozygosity H_p_ for each simulation, as the mean homozygosity H_i_ across all individuals in all subpopulations (Fig. 1G). To tease apart different mechanisms that could affect H_p_ (Fig. 1a–d), and to separate the direct effect of polyandry from the effect of female sperm limitation caused by inbreeding depression in sperm number, we ran simulations with fixed monandry by fixing female remating rate to τ = 1. We calculated the total variances in female (*V_f_*) and male (*V_m_*) reproductive success across all subpopulations, and then calculated the effective population size attributable to these variances (hereafter *N_ev_*) using the approximation (Falconer and Mackay 1996):

(9) $$N_{ev}=\frac{8N}{V_{m}\;+\;V_{f}\;+\;4},$$

where *N* is the total population size. *N_ev_* thereby captures the expected effects of the variances in both sexes’ reproductive success on the rate of increase of inbreeding across generations and hence on observed H_p_. However, observed H_p_ could differ from that expected solely given *V_f_* and *V_m_*, and hence *N_ev_*, if variation in male reproductive success is nonrandom with respect to male homozygosity and homozygosity is heritable. To quantify such effects, we calculated the correlation between male H_i_ and the total number of offspring each male sired, and the slope of the father–offspring regression in H_i_ across all such individuals.

Finally, to evaluate the potential impact of the evolving mating system on population fitness, for each simulation we calculated mean female fertility, measured as the mean number of offspring produced by each female before density‐dependent selection was applied. To avoid confounding effects due to extinctions, we set a moderately high female fecundity (*R* = 8) and density regulation that generates stable population dynamics. Consequently, even substantial decreases in female fertility do not constrain adult population size in our simulations. However, we interpret female fertility as a proxy of potential population growth rate and buffer against demographic stochasticity, and hence as a proxy of population fitness.

Each simulation was run for 10,000 generations to reach evolutionary equilibria, and replicated 50 times. Model source code is available in the Dryad Digital Repository. Although our primary simulations consider the case where inbreeding depression affects sperm number (*s*), we ran additional simulations to test whether emergent evolutionary dynamics differ if inbreeding depression instead affects sperm mortality rate (μ) or the “cost‐free” resources available for allocation to sperm (ρ_0_; Supporting Information S7). To verify that our results are not contingent on assuming a trade‐off between *s* and μ, we also ran equivalent simulations with a modified model that did not include μ (Supporting Information S3), and thereby considered *s* as the only evolving male trait.

## Results

### EVOLUTION OF COSTLY POLYANDRY

**Figure 2:**
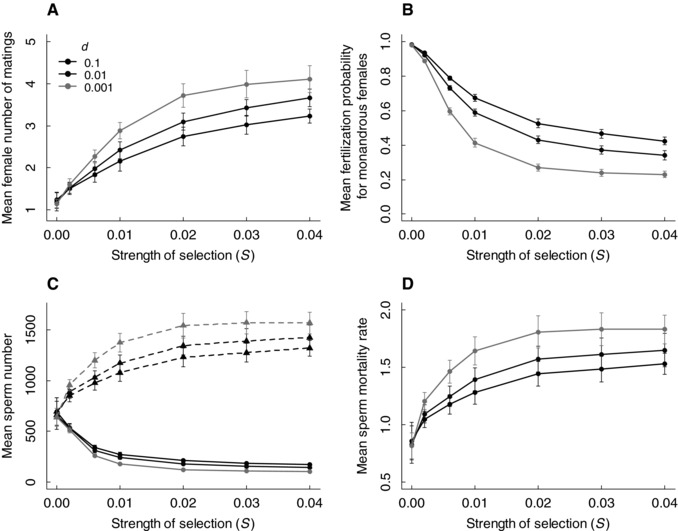
Polyandry evolved with increasing inbreeding depression in sperm number due to increased female sperm limitation. (A) Number of female matings per fertilization event (1/τ) and hence the degree of polyandry, (B) fertilization probability for a monandrous female (Φ), (C) phenotypic (*s*; solid lines and circles) and genotypic (*g_s_*; dashed lines and triangles) sperm number, and (D) phenotypic sperm mortality rate (μ) that evolved at seven strengths of selection against deleterious mutations (*S* = 0, 0.002, 0.006, 0.01, 0.02, 0.03, 0.04) and three dispersal probabilities (*d* = 0.1, black; 0.01, dark gray; 0.001, light gray). Data show the mean values across all individuals at generation 10,000 averaged across 50 replicates. Bars indicate twice the standard deviation around the replicate means.

As hypothesized (Fig. 1A–D), female remating rate, and hence polyandry, evolved in response to inbreeding depression in sperm number (Fig. 2A), despite the direct cost of polyandry that reduced female survival. The stronger the inbreeding depression, as determined by the strength of selection against deleterious mutations (*S*) and by the population‐wide degree of inbreeding (controlled by dispersal probability), the greater the evolutionary increase in polyandry. This increase was caused by the decreased mean fertilization probability for monandrous females (Fig. 2B). Females consequently evolved to mate multiply to ensure fertility given the presence of inbred males with low sperm number.

Imposing inbreeding depression in sperm number also caused evolutionary changes in both sperm traits. Specifically, the genotypic value for sperm number increased with increasing inbreeding depression, causing a smaller reduction in phenotypic sperm number than that expected simply given the imposed magnitude of inbreeding depression (Fig. 2C). The phenotypic (and genotypic) value for sperm mortality rate also increased with increasing inbreeding depression (Fig. 2D) as a consequence of the trade‐off with sperm number, thus contributing to the reduction in mean fertilization probability.

Quantitatively, evolution of polyandry and sperm traits depended on the cost of sperm; greater polyandry evolved given greater sperm cost (Supporting Information S2). However, key results remained qualitatively similar when sperm mortality rate was excluded from the model, showing that inbreeding depression in one sperm trait can drive polyandry evolution without necessarily requiring direct trade‐offs with other sperm traits (Supporting Information S3).

### FEED‐BACK FROM SPERM COMPETITION TO POLYANDRY

**Figure 3:**
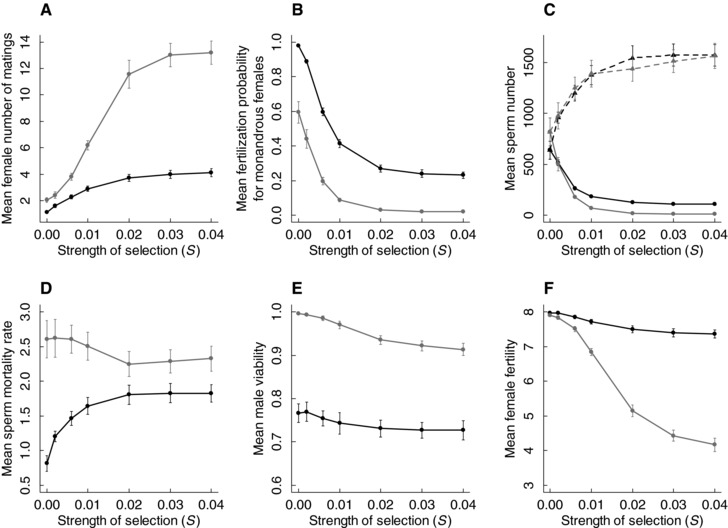
Selection on sperm traits exerted by sperm competition counteracted the reduction in female fertilization probability caused by inbreeding depression in sperm number, causing lower female remating rate to evolve with (black) versus without (gray) sperm competition. (A) Number of female matings per fertilization event (1/τ) and hence the degree of polyandry, (B) fertilization probability for a monandrous female (Φ), (C) phenotypic (*s*; solid lines and circles) and genotypic (*g_s_*; dashed lines and triangles) sperm number, (D) phenotypic sperm mortality rate (μ), (E) male viability and (F) female fertility (i.e., mean number of offspring produced per female) at seven strengths of selection against deleterious mutations (*S* = 0, 0.002, 0.006, 0.01, 0.02, 0.03, 0.04) given dispersal probability, *d* = 0.001. Data show the mean values across all individuals at generation 10,000, averaged across 50 replicates. Bars indicate twice the standard deviation around the replicate means.

Simulations where sperm competition was excluded showed that, as hypothesized (Fig. 1A–F), sperm competition resulting from evolving polyandry, and consequent evolution of sperm traits, played a central role in shaping polyandry evolution given inbreeding depression in sperm number (Fig. 3). Surprisingly, in simulations without sperm competition, females evolved much higher remating rates due to decreased mean fertilization probability with increasing inbreeding depression (Fig. 3A and B). Low fertilization probability arose because, without the selection stemming from sperm competition, genotypic values of male sperm traits did not evolve sufficiently to compensate for inbreeding depression in sperm number. Although with increasing strength of selection against deleterious mutations, the mean genotypic value of sperm number increased, and sperm mortality rate decreased (Fig. 3C and D), sperm mortality rate evolved to much higher values compared to simulations that allowed sperm competition (Fig. 3D). Overall, males invested less in sperm traits and more in their own survival (Fig. 3E), thereby reducing sperm viability and hence fertilization probability. This is because in the absence of sperm competition, male survival, and hence the opportunity to participate in multiple female fertilization events, becomes the main factor determining male fitness.

Importantly, even given the increased female remating rate resulting from low male investment in sperm traits, the mean number of offspring produced per female was substantially lower when sperm competition was excluded, especially given strong inbreeding depression (Fig. 3F). This was caused by both decreased female survival, due to the cost of increased remating rates, and increased female infertility (Supporting Information S4). These results indicate that sperm competition, which itself results from polyandry, can partially mitigate the negative effects of inbreeding depression in sperm number on female reproductive success.

### FEED‐BACK FROM THE EVOLVED MATING SYSTEM TO POPULATION‐WIDE HOMOZYGOSITY

**Figure 4:**
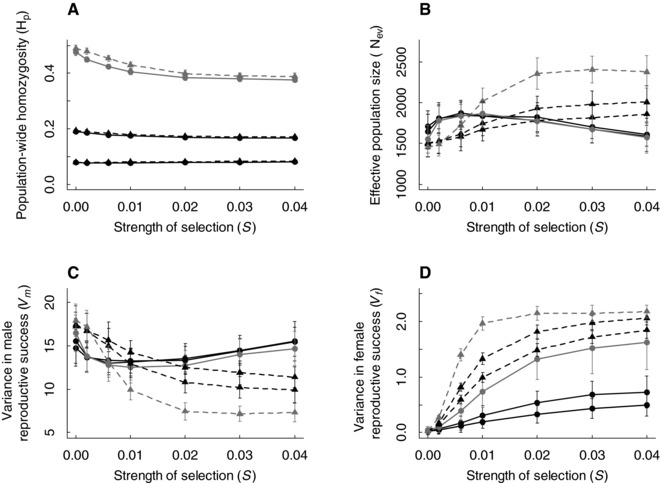
Increasing inbreeding depression in sperm number decreased population‐wide homozygosity (H_p_) due to interacting effects of polyandry, resulting sperm competition, and inbreeding depression. (A) H_p_; (B) effective population size (*N_ev_*); (C) variance in male reproductive success (*V_m_*); and (D) variance in female reproductive success (*V_f_*) at seven strengths of selection against deleterious mutations (*S* = 0, 0.002, 0.006, 0.01, 0.02, 0.03, 0.04) and three dispersal probabilities (*d* = 0.1, black; 0.01, dark gray; 0.001, light gray). In all panels, circles and solid lines indicate evolving polyandry, while triangles and dashed lines indicate fixed monandry. Data show (A) the mean H_p_, (B) the mean *N_ev_*, and (C and D) the mean variances at generation 10,000 across 50 replicate simulations. Bars indicate twice the standard deviation around the replicate means; in (A) bars are present but very small. Note that the *y*‐axis scales differ between (C) and (D).

Alongside the expected effects of dispersal probability, population‐wide homozygosity measured across the neutral loci (H_p_) decreased with increasing strength of selection against deleterious mutations (*S*) at intermediate and low dispersal probabilities (Fig. 4A). In contrast, there was no change in H_p_ with increasing *S* given higher dispersal probability (Fig. 4A). Further, within each level of *S*, H_p_ was slightly lower when polyandry was allowed to evolve than when all females were fixed to be monandrous (Fig. 4A). This indicates that evolution of polyandry reduced H_p_, especially given low dispersal probability (Fig. 4A).

Multiple mechanisms could contribute to the observed decrease in H_p_ with increasing *S* and resulting evolution of polyandry, stemming from interacting effects of sperm competition and inbreeding depression in sperm number (Fig. 1a–d). Further comparisons of simulations with fixed monandry versus evolving polyandry serve to elucidate these mechanisms (Fig. 4B–D, solid vs. dashed lines).

Given fixed monandry (i.e., no sperm competition), increasing *S* caused a reduction in H_p_ (Fig. 4A, dashed lines). The variance in male reproductive success (*V_m_*) decreased substantially with increasing inbreeding depression, thereby reducing the total variance in reproductive success, despite a slight increase in the variance in female reproductive success (*V_f_*) (Fig. 4C–D, dashed lines). The decrease in *V_m_* was primarily due to the substantial reduction in variance in sperm number caused by increasing inbreeding depression (Supporting Information S5). Conversely, the increase in *V_f_* was due to the decreased mean fertilization probability given increasing inbreeding depression in sperm number (Supporting Information S6), and hence increased chance of infertility and consequent reproductive failure of females that mated with inbred males (Fig. 1A). The overall reduction in variance in reproductive success thus increased *N_ev_* (Fig. 4B) and consequently reduced H_p_.

With evolving polyandry, H_p_ again decreased with increasing *S* given intermediate and low dispersal probabilities (Fig. 4A, solid lines). However, H_p_ was lower given evolving polyandry than with fixed monandry (Fig. 4A). Given *S* ≤ 0.006, both *V_m_* and *V_f_* were reduced compared to fixed monandry (Fig. 4C and D), causing higher *N_ev_*, and hence lower H_p_, given evolving polyandry (Fig. 4A and B). With polyandry, even males whose sperm traits mean that they could not fertilize a monandrous female have some chance to gain some paternity (Bocedi and Reid 2016), meaning that paternity is more evenly distributed across males (Fig. 1b). Additionally, the chance of female reproductive failure due to sperm limitation is reduced (as females evolve to mate multiply to ensure fertility; Fig. 1b).

In contrast to the decrease in *V_m_* observed given fixed monandry, *V_m_* increased given evolving polyandry and *S* ≥ 0.01 (Fig. 4C, solid lines). As *S* increased, increased sperm limitation caused increased polyandry, and hence increased sperm competition. This in turn caused further evolutionary increases in genotypic values for sperm number and mortality rate (Fig. 2). The interaction between changes in male genotypic distributions and *S* increased the variance in both sperm number and mortality rate (Supporting Information S5), causing higher *V_m_*. The increase in *V_m_* caused *N_ev_* to decrease (Fig. 4B).

**Figure 5:**
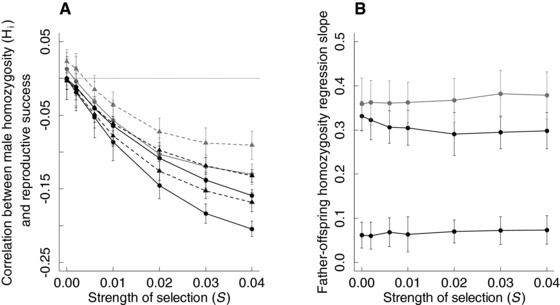
Increasing inbreeding depression in sperm number strengthened the negative correlation between a male's homozygosity and its reproductive success, thus decreasing population‐wide homozygosity because of the heritability of homozygosity. (A) Correlation between a male's homozygosity across neutral loci (H_i_) and his reproductive success (number of offspring sired), and (B) slope of the regression of offspring homozygosity H_i_ on father H_i_, at seven strengths of selection against deleterious mutations (*S* = 0, 0.002, 0.006, 0.01, 0.02, 0.03, 0.04,) and three dispersal probabilities (*d* = 0.1, black; 0.01, dark gray; 0.001, light gray). In (A), circles and solid lines indicate evolving polyandry, triangles and dashed lines indicate fixed monandry, and the dotted line demarcates zero correlation. Correlations are calculated across males in each subpopulation across generations, and then averaged across subpopulations. In (B), slopes are shown for simulations with evolving polyandry; slopes from simulations with fixed monandry were quantitatively similar. Regressions are computed across all offspring across all subpopulations at generation 10,000. In both panels, data show correlations and regression slopes averaged across 50 replicate simulations. Bars indicate twice the standard deviation around the replicate means.

However, despite reduced *N_ev_*, H_p_ did not increase (Fig. 4A). This is explained by a negative correlation between a male's own homozygosity H_i_ and his reproductive success, which was stronger with greater *S* (Figs. 1d and 5A). Less homozygous males were therefore more successful sires. Because homozygosity was heritable, as shown by the positive slope of the father–offspring regression in H_i_ (Fig. 5B), offspring of less homozygous males were less homozygous themselves, thereby decreasing H_p_. The father–offspring regression slope was greater given lower dispersal probability, explaining the larger reduction in H_p_ at lower dispersal. This is expected because the heritability of homozygosity stems from unequal allele frequencies, which is expected to be greater with limited gene flow among populations (Fromhage et al. 2009; Nietlisbach et al. 2016). Overall, therefore, evolving polyandry resulting from strong inbreeding depression in a key male sperm trait generates two contrasting mechanisms, increased *V_m_* and higher reproductive success of heterozygous males (Fig. 1c–d), whose net effect is to reduce H_p_.

Although we observed substantial purging of deleterious mutations with increasing *S*, such purging was not sufficient to eliminate inbreeding depression in sperm number, and therefore to eliminate female sperm limitation and consequent selection for polyandry (Supporting Information S1).

### EFFECT OF THE EVOLVING MATING SYSTEM ON FEMALE FERTILITY

**Figure 6:**
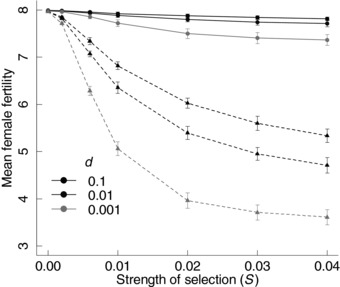
Evolving polyandry buffered the negative effect of inbreeding depression in sperm number on female reproductive success. Mean female fertility (i.e., mean female's number of offspring) at seven strengths of selection against deleterious mutations (*S* = 0, 0.002, 0.006, 0.01, 0.02, 0.03, 0.04) and three dispersal probabilities (*d* = 0.1, black; 0.01, dark gray; 0.001, light gray) from simulations with evolving polyandry (solid circles and lines) versus fixed monandry (triangles and dashed lines). Data show the mean values across all individuals at generation 10,000, averaged across 50 replicates. Bars indicate twice the standard deviation around the replicate means.

The evolving mating system affected the mean number of offspring produced per female (female fertility; Fig. 6). With evolving polyandry, there was no, or only slight, reduction in mean female fertility with increasing *S* (Fig. 6A, solid lines). Conversely, with fixed monandry mean female fertility decreased substantially with increasing *S* (Fig. 6A, dashed lines). In both cases, lower dispersal probability led to lower mean female fertility, with a much stronger effect given fixed monandry. Thus, because female fertility can be interpreted as a proxy for population fitness, evolving polyandry has the potential to buffer the deleterious effect of inbreeding depression in sperm traits on overall population fitness.

### INBREEDING DEPRESSION IN DIFFERENT MALE TRAITS

The effect of inbreeding depression on polyandry evolution, and hence on male traits, H_p_ and female fertility, was similar when inbreeding depression increased sperm mortality rate rather than decreased sperm number (Supporting Information S7). However, when inbreeding depression instead reduced the “cost‐free” resources available for sperm allocation (ρ_0_), lower polyandry evolved, and H_p_ and female fertility changed little with increasing *S*, even though male viability was substantially reduced (Supporting Information S7). Therefore, with inbreeding depression in resources available for sperm allocation, males did not invest less in sperm even though this strongly reduced their viability. Consequently, female sperm limitation did not increase with increasing *S* and there was very little selection for polyandry.

## Discussion

Feed‐backs between evolving components of mating systems and their genetic environment could have profound consequences for the evolutionary dynamics of mating systems themselves, and also for evolution of other life‐history traits and resulting population viability (Holman and Kokko 2013). However, such feed‐backs are rarely explicitly considered in the context of understanding the evolutionary causes and consequences of nonselfing mating systems. Our model shows that these feed‐backs can link evolution of costly polyandry and consequent sperm competition with dynamics of reproductive success, inbreeding, and homozygosity arising in spatially structured populations (Fig. 1), and clarifies the mechanisms underlying such feed‐backs.

### INBREEDING AS A DRIVER OF POLYANDRY EVOLUTION

Evolution of costly polyandry remains a conundrum, but is widely hypothesized to be driven by inbreeding risk arising in populations where relatives interact, and by resulting indirect selection stemming from inbreeding depression in offspring viability. Specifically, polyandry could evolve to allow females to actively avoid inbreeding through mate choice, by mating with and/or biasing paternity toward less related males (Pusey and Wolf 1996; Jennions and Petrie 2000; Blomqvist et al. 2002; Tregenza and Wedell 2002; Pizzari et al. 2004b; Fitzpatrick and Evans 2014; While et al. 2014). However, a recent model showed that evolution of polyandry to avoid inbreeding through precopulatory mate choice might require restricted conditions, including low direct cost of polyandry, strong inbreeding depression, and highly constrained initial mate availability (Duthie et al. 2016). Moreover, although polyandry is commonly observed to co‐occur with inbreeding, it does not always lead to inbreeding avoidance, even given substantial inbreeding depression in offspring viability (Jennions et al. 2004; Billing et al. 2012; Slatyer et al. 2012; Reid et al. 2015).

In contrast, our model supports the hypothesis (Fig. 1A–D) that costly polyandry can readily evolve to ensure female fertility given sperm limitation caused by inbreeding depression in sperm traits. Here, polyandry evolves because it allows females to mitigate the consequences of mating with inbred males, rather than directly avoid the consequences of inbreeding with related males. The occurrence of inbreeding within a population can therefore drive evolution of polyandry without need for mechanisms of precopulatory or postcopulatory inbreeding avoidance.

Our results imply that increased polyandry might evolve in viscous, small, or fragmented populations where inbreeding occurs and inbreeding depression in male gametic traits is strong. This situation might apply to diverse species and populations, for example, because the population has recently become inbred due to habitat fragmentation and costly dispersal, and inbreeding avoidance mechanisms have not yet evolved or cannot be enacted. Indeed, inbreeding depression in diverse male gametic traits, including sperm number, viability, and competitiveness, is widely observed (e.g., Zajitschek et al. 2009; Michalczyk et al. 2010; Fox et al. 2012; Gasparini et al. 2013; Losdat et al. 2014), although further evidence from wild populations would be valuable. Moreover, purging might be too weak to completely eliminate mutation load, even given strong selection against deleterious mutations (Supporting Information S1). Further theory is needed to fully understand how the combination of biparental inbreeding (Hedrick 1994; Wang et al. 1999; Porcher and Lande 2016), sexual selection (Whitlock and Agrawal 2009; Arbuthnott and Rundle 2012; Almbro and Simmons 2014; Lumley et al. 2015), and natural selection combine to purge deleterious mutations in small, spatially structured populations, and how this might impact polyandry evolution. In our model, despite purging, greater polyandry evolved given stronger selection against deleterious mutations and little dispersal, and hence more inbreeding and inbreeding depression in male traits. This implies that degrees of polyandry and inbreeding might be correlated across populations, but not necessarily be directly causally linked through inbreeding avoidance.

### THE ROLE OF SPERM COMPETITION

Our model also shows that, as hypothesized (Fig. 1B–F), the direct effect of inbreeding depression in male traits on polyandry evolution interacted with resulting sperm competition, causing feed‐back dynamics that affected evolution of sperm traits and polyandry. We previously showed that, in the absence of inbreeding depression, sperm competition resulting from initial polyandry can exacerbate female sperm limitation and hence feed‐back to cause further evolution and maintenance of costly polyandry (Bocedi and Reid 2016). Our current model demonstrates a more complex role of sperm competition in driving mating system evolution. Given the additional challenge of inbreeding depression in sperm traits, sperm competition drove further evolutionary changes in sperm traits that compensated for reduced male fertilization efficiency due to inbreeding depression (Figs. 2 and 3). Thus, on the one hand, sperm competition can feed back to drive polyandry evolution (Bocedi and Reid 2016). On the other hand, the direct selection that sperm competition exerts on male sperm competitive ability causes evolution of sperm traits that buffers the negative effect of inbreeding depression on female sperm limitation and hence fertility, impeding further evolution of costly polyandry. This suggests that, given inbreeding depression in sperm traits, by causing sperm competition polyandry has the potential to increase population viability by counteracting negative effects of inbreeding depression on male and female fertility and hence reproductive success (Figs. 3 and 6). This opens an intriguing possibility that polyandry evolution might act as a form of evolutionary rescue (e.g., Gonzalez et al. 2013) that would allow a population to recover from a fitness decrease due to a sudden increase in inbreeding rate (e.g., because of recent habitat fragmentation and increased cost of dispersal).

### FEED‐BACK BETWEEN MATING SYSTEM AND POPULATION‐WIDE HOMOZYGOSITY

As hypothesized (Fig. 1G, H, and a–d), the evolving mating system comprising polyandry and sperm traits can feed back to reduce population‐wide homozygosity (Fig. 4), and ameliorate the negative effect of inbreeding depression on female fertility (Figs. 3B and 6). However, multiple, sometimes opposing, mechanisms acted simultaneously to generate a small overall net reduction in population‐wide homozygosity. Especially at high levels of selection against deleterious mutations, the increase in polyandry increased the variance in male reproductive success, thereby preventing a further reduction in population‐wide homozygosity. Notably however, even when the variance in male reproductive success increased, population‐wide homozygosity did not increase further because of the negative correlation between male homozygosity and siring success and the heritability of homozygosity.

Such feed‐back dynamics might not only affect evolution of the focal mating system components, but also affect wider aspects of population ecology and evolutionary dynamics. For example, the resulting reduction in population‐wide homozygosity could relax selection for active inbreeding avoidance through pre‐ and/or postcopulatory mate choice, or through sex‐biased dispersal (Gandon 1999; Guillaume and Perrin 2006; Henry et al. 2016), and hence affect evolution of other mating system and life‐history traits which are driven by population relatedness structure, inbreeding risk, and inbreeding depression.

Further, polyandry and the occurrence of inbreeding could be intrinsically linked because, by producing maternal half‐sibs instead of full‐sibs, polyandrous females could reduce sib–sib inbreeding among their offspring, resulting in less inbred grand–offspring (Cornell and Tregenza 2007; Michalczyk et al. 2011; Power and Holman 2014). However, selection stemming from production of half‐sibs rather than full‐sibs might be very weak, except when a species’ ecology means that sib mating is common, such as when resource patches are colonized by single already‐mated females (Cornell and Tregenza 2007). In our model, where populations are spatially structured, the effect of polyandry itself in reducing population‐wide homozygosity, and hence the overall level of inbreeding, was rather small compared to the effect of inbreeding depression across levels of selection (Fig. 4).

### ASSUMPTIONS AND PERSPECTIVES

Our current aim was to test hypotheses regarding the consequences of female mating with inbred males, and resulting feed‐backs that could drive evolution of costly polyandry in the context of inbreeding (Fig. 1), as opposed to testing hypotheses regarding active inbreeding avoidance. Consequently, we did not model inbreeding depression in offspring viability, although this is often one primary cost of inbreeding and hence driver of inbreeding avoidance (Keller and Waller 2002). However, if there was substantial inbreeding depression in offspring viability as well as in male sperm traits, stronger selection for inbreeding avoidance through mate choice might be expected, for example, through polyandry and associated pre‐ or postcopulatory mechanisms (Duthie and Reid 2016; Duthie et al. 2016), or through sex‐biased dispersal (Gandon 1999; Guillaume and Perrin 2009; Henry et al. 2016). The evolution of such strategies could decrease the overall population‐wide level of inbreeding and hence reduce expression of inbreeding depression in sperm traits and consequent female sperm limitation and resulting selection for polyandry. Which mechanism would prevail will depend on the relative strength of inbreeding depression in male gametic traits versus offspring viability, on how these components of inbreeding depression affect individual fitness, and on the costs (including opportunity costs) of different strategies, including the many potential costs of dispersal (e.g., Bonte et al. 2012).

Our model also assumes that females cannot asses the inbreeding level, and hence the fertility, of potential mates, and therefore cannot make any active choice to avoid more inbred and less fertile males. In reality, there is evidence that secondary sexual characters can indicate individual heterozygosity or coefficient of inbreeding (e.g., Van Oosterhout et al. 2003; Reid et al. 2005; Kempenaers 2007; Herdegen et al. 2014; Ferrer et al. 2015), and indicate sperm traits and/or male fertilization ability (e.g., Wagner and Harper 2003; Helfenstein et al. 2010; Kekäläinen et al. 2014; Mehlis et al. 2015; but see Pizzari et al. 2004a; Kvarnemo and Simmons 2013; Lüpold et al. 2014). In these cases, direct female choice might be a more efficient route than polyandry to avoid mating with inbred males, at least when mate choice is not constrained by other factors. However, very few studies have investigated the full pathway linking secondary sexual characters with fertilization efficiency through heterozygosity (Zajitschek and Brooks 2010).

We implemented a genetically explicit model of inbreeding depression, where mutation load, inbreeding load, and consequent inbreeding depression can evolve through purging and fixation of deleterious mutations. However, the loci at which deleterious mutations occur are different from the loci that determine the genotypic value for the trait affected by inbreeding depression. Therefore, as done in other models (e.g., Porcher and Lande 2005; Guillaume and Perrin 2006; Roze and Rousset 2009; Duthie and Reid 2016; Henry et al. 2016), we set the dominance and strength of selection against deleterious mutations, instead of allowing these properties to emerge from the model. Indeed, how inbreeding depression evolves for polygenic sexually selected traits, which can be subject to opposing natural and sexual selection, is an exciting open question, and the genetic architectures and magnitudes of inbreeding depression arising for different traits are not easily predictable.

Despite these open questions, our model demonstrates a novel and underappreciated way by which inbreeding could select for polyandry through postcopulatory processes, and feed back to affect population‐wide homozygosity. This mechanism and other hypothesized mechanisms linking inbreeding and polyandry are not necessarily mutually exclusive. They could all contribute to the overall evolutionary dynamics of mating systems, with their relative importance depending on species’ life histories and underlying genetic architectures. Overall, future fully genetically explicit models that consider a dynamic genetic environment and evolving dominance and inbreeding depression in different components of male and female fitness, alongside other postulated mechanisms of inbreeding avoidance, including dispersal and/or pre‐ or postcopulatory female choice for male heterozygosity, should serve to elucidate how these different mechanisms collectively shape the evolutionary dynamics of reproductive systems involving inbreeding, and how these feed back to population ecological and evolutionary dynamics.

Associate Editor: B. Hollis

Handling Editor: M. Servedio

## Supporting information


**Table S1**. Model variables and parameters.
S1. Purging of deleterious mutations and evolution of inbreeding depression
S2. Effect of costs of polyandry and sperm on polyandry evolution
S3. Polyandry evolution in the absence of a trade‐off between sperm number and sperm mortality rate
S4. Determinants of female fertility in simulations with and without sperm competition
S5. Among‐male variance in sperm trait phenotypes
S6. Fertilization probability for monandrous females under evolving polyandry versus fixed monandry
S7. Inbreeding depression in different male traits
