# Supplementary material for: Feed‐backs among inbreeding, inbreeding depression in sperm traits, and sperm competition can drive evolution of costly polyandry
Source: Evolution. 2017 Nov 13;71(12):2786–802. doi: 10.1111/evo.13363 (PMC5765454; doi:10.1111/evo.13363)
Supplement: Supplementary file 1 — Table S1. Model variables and parameters. S1. Purging of deleterious mutations and evolution of inbreeding depression S2. Effect of costs of polyandry and sperm on polyandry evolution S3. Polyandry evolution in the absence of a trade‐off between sperm number and sperm mortality rate S4. Determinants of female fertility in simulations with and without sperm competition S5. Among‐male variance in sperm trait phenotypes S6. Fertilization probability for monandrous females under evolving polyandry versus fixed monandry S7. Inbreeding depression in different male traits [file EVO-71-2786-s001.docx]

**Feed-backs among inbreeding, inbreeding depression in sperm traits and sperm competition can drive evolution of costly polyandry**

**Supplementary Information**

[**Table S1. Model variables and parameters 2**](#_Toc483231542)

[**S1. Purging of deleterious mutations and evolution of inbreeding depression 4**](#_Toc483231543)

[**S2. Effect of costs of polyandry and sperm on polyandry evolution 7**](#_Toc483231544)

[**S3. Polyandry evolution in the absence of a trade-off between sperm number and sperm mortality rate 10**](#_Toc483231545)

[**S4. Determinants of female fertility in simulations with and without sperm competition 13**](#_Toc483231546)

[**S5. Among-male variance in sperm trait phenotypes. 14**](#_Toc483231547)

[**S6. Fertilization probability for monandrous females under evolving polyandry versus fixed monandry 16**](#_Toc483231548)

[**S7. Inbreeding depression in different male traits. 17**](#_Toc483231549)

[**References 23**](#_Toc483231550)

# Table S1. Model variables and parameters

| **Variables** | **Description** | **Parameter values** |
| --- | --- | --- |
| *K* | Carrying capacity per cell (sub-population) | 160 individuals |
| *d* | Dispersal probability | 0.1, 0.01, 0.001 |
| *R* | Female fecundity | 8 |
| *r* | Fertilization rate | 0.015 (corresponding to ~ 300 sperm cells for Φ = 0.99) |
|  | Number of diploid loci for each trait | 20 |
| *φ* | Fertilization probability of an individual male |  |
| *Ζ* | Total number of viable sperm available to a female^†^ |  |
| Φ | Egg fertilization probability |  |
| *ψ* | Individual survival probability |  |
| **Inbreeding and inbreeding depression** | |  |
|  | Number of diploid loci carrying deleterious mutations | 1000 |
| Θ | Individual number of loci homozygous for deleterious mutations |  |
| θ | Individual number of loci heterozygous for deleterious mutations |  |
| *S* | Strength of selection against deleterious homozygous mutations | 0, 0.002, 0.006, 0.01, 0.02, 0.03, 0.04 |
| *h* | Dominance coefficient of deleterious mutations | 0 |
|  | Number of neutral diploid loci | 1000 |
|  | Initial real uniform distribution for the neutral loci | U[-1000.0,1000.0] |
| H_i_ | Individual neutral homozygosity (number of homozygote neutral loci / 1000) |  |
| H_p_ | Population-wide homozygosity (mean H_i_ across all individuals across all sub-populations) |  |
| **Mutations** |  |  |
|  | Mutation probability (per allele per generation) | 0.001 |
|  | Mean mutational effect for evolving traits | 0.0 |
|  | Variance in mutational effects for evolving traits | 0.1(*σ^2^_τ,0_* / 40); 0.1(*σ^2^_s,0_* / 40); 0.1(*σ^2^_µ,0_* / 40) |
| **Traits** |  |  |
| *g_τ_* | Female re-mating interval (genotypic value) |  |
| *g_s_* | Sperm number (genotypic value) |  |
| *g_µ_* | Sperm mortality rate (genotypic value) |  |
| *τ* | Female re-mating interval (phenotypic value) | 0.01 ≤ *τ* ≤ 1.0 (i.e., 1 ≤ number of mating per egg ≤ 100) |
| *s* | Sperm number (phenotypic value)^‡^ | *s* ≥ 1.0 |
| *µ* | Sperm mortality rate (phenotypic value)^‡^ | *µ* ≥ 10^-10^ |
| **Trait Initialization** | |  |
|  | Initial genotypic mean for female re-mating interval | 0.5 |
|  | Initial genotypic mean for sperm number | 500 |
|  | Initial genotypic mean for sperm mortality rate | 0.5 |
| *σ^2^_τ,0_* | Initial genotypic variance for female re-mating interval | 0.1 |
| *σ^2^_s,0_* | Initial genotypic variance for sperm number | 10000.0 |
| *σ^2^_µ,0_* | Initial genotypic variance for sperm mortality rate | 1.0 |
| **Costs and trade-off** | |  |
| *ω^2^_f_* | Strength of direct selection (cost) for female multiple mating | 1.28×10^5^ |
| *ω^2^_m_* | Strength of direct selection against investment in sperm (i.e. cost of sperm) | 1.0 |
| *ρ_0_* | Maximum amount of resources available to allocate to sperm without incurring a survival cost | 1.0 |
| *ρ* | Male resource allocation to sperm |  |
| *β* | Scaling parameter determining the cost of a single sperm cell | 0.001, 0.002, 0.003 |

### ^†^The total number of viable sperm (*Z*) available to a female at the time of fertilization (*t*) may include sperm from previous fertilization events.

### ^‡^We assume males cannot produce < 1 sperm cell, and limit *µ* to > 10^-10^ to avoid numerical errors given *µ* = 0.

# S1. Purging of deleterious mutations and evolution of inbreeding depression

In general, the occurrence of inbreeding is expected to cause purging of deleterious recessive mutations and thus reduce inbreeding depression, and thereby reduce selection against inbreeding and hence for all forms of inbreeding avoidance (Lande et al. 1994; Wang et al. 1999; Crnokrak and Barrett 2002). However, while such purging can occur given self-fertilization, the degree of purging that might arise in non-selfing mating systems is less clear (Hedrick 1994; Duthie and Reid 2016; Porcher and Lande 2016)

In our model, the frequency of deleterious mutations after 10,000 generation decreased with increasing strength of selection *S*, providing evidence of greater purging with increased *S* (Fig. S1.1A). Specifically, purging arose because, as *S* increased, males that were more inbred and with a higher mutation load had lower reproductive success (Fig. 5A), thus reducing the frequency of deleterious mutations. The degree of purging was slightly smaller given evolving polyandry than given fixed monandry (Fig. S1.1B). This is because polyandry reduced mean population-wide homozygosity (H_p_) below that observed given monandry (Fig. 4B). Deleterious mutations were consequently less visible to selection. However, the difference in the degree of purging between simulations with and without evolving polyandry was very small.

However, despite the increased purging, the expressed magnitude of inbreeding depression still increased with increasing *S* (Fig. S1.2). The expressed mutational damage (intercept of regressions in Fig. S1.2; Morton et al. 1956) increased with increasing *S*, and was greater at lower dispersal probability, indicating greater fixation of deleterious mutations due to drift (Wang et al. 1999). The slope of inbreeding depression became steeper with increasing *S* at high and moderate dispersal probability (*d* = 0.1 and 0.01), but less steep at low dispersal probability (*d* = 0.001), indicating a greater effect of purging in the latter case. This is to be expected, as with limited dispersal, the degree of inbreeding is higher, thus causing greater exposure of deleterious recessive mutations to selection (Wang et al. 1999). Thus, in our model, although substantial purging of deleterious recessive mutations occurred with increasing *S*, purging was not sufficient to eliminate inbreeding depression in sperm traits, and hence female sperm limitation and selection for polyandry. Moreover, evolution of polyandry had only a slight effect on the level of purging.


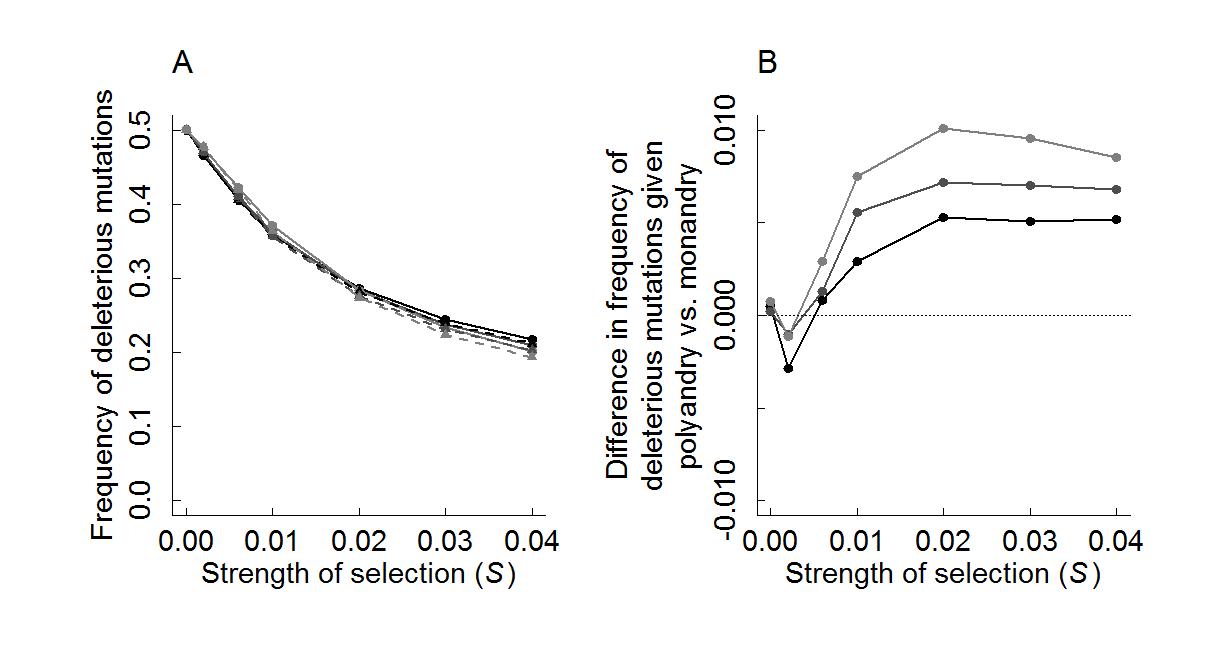


**Figure S1.1.** The frequency of deleterious recessive mutations affecting sperm number decreased with increasing strength of selection against such mutations and was marginally higher given evolving polyandry. (A) Frequency of deleterious mutations given evolving polyandry (circles and solid lines) and fixed monandry (triangles and dashed lines), and (B) the difference between the two frequencies, at seven strengths of selection against deleterious mutations (*S* = 0, 0.002, 0.006, 0.01, 0.02, 0.03, 0.04) and three dispersal probabilities (*d* = 0.1, black; 0.01, dark grey; 0.001, light grey). Data show (A) the mean frequency at generation 10,000 across 50 replicate simulations and (B) the differences among replicate mean frequencies. On (B) the dotted line demarcates zero difference. Bars indicating twice the standard deviation around the replicate means are present in panel A but not visible because they are very small.


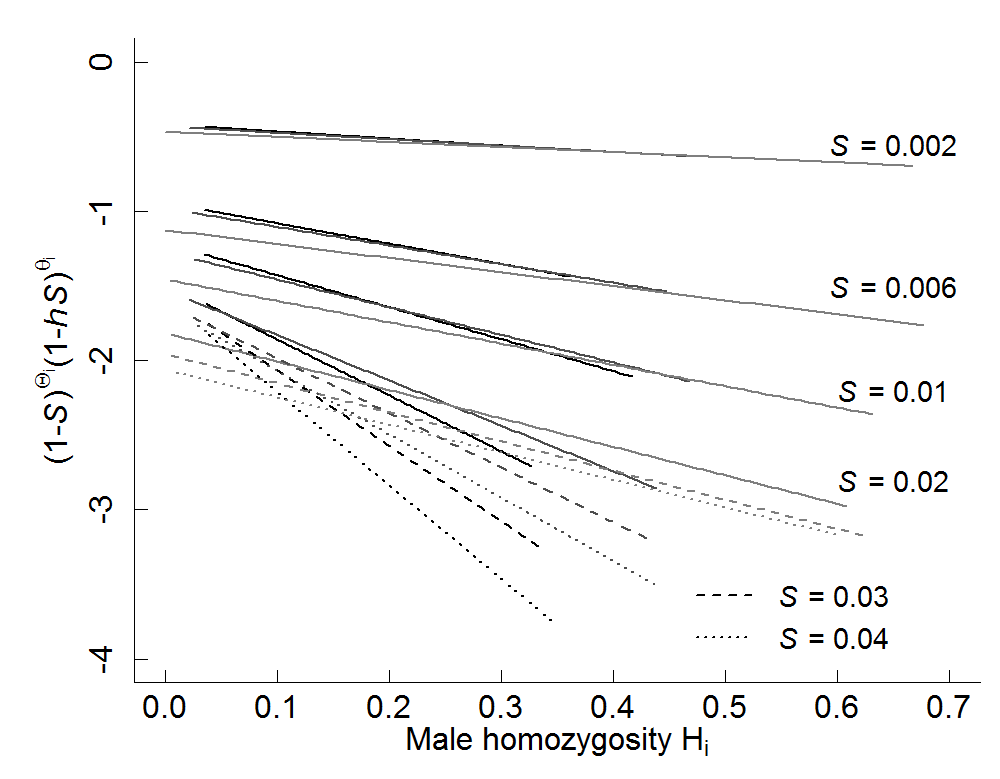


**Figure S1.2.** Inbreeding depression in sperm number, given evolving polyandry, increased with increasing strength of selection against deleterious mutations (*S*), and depended on dispersal probability (*d* = 0.1, black; 0.01, dark grey; 0.001, light grey). Inbreeding depression is calculated as the slope of the regression of the natural logarithm of each male’s effect of deleterious mutations $\left(1-S\right)^{\text{Θ}}\left(1-hS\right)^{\text{θ}}$ (cf. eqn. 1) on his homozygosity (H_i_), at generation 10,000 across 50 replicates. For clarity, dashed lines represent *S* = 0.03, while dotted lines represent *S* = 0.04.

# S2. Effect of costs of polyandry and sperm on polyandry evolution

*Cost of female multiple mating*

The magnitude of the direct cost of female multiple mating will inevitably impact the evolutionary dynamics of polyandry; polyandry will clearly not evolve when the direct cost exceeds any possible benefit. We previously explored the effect of varying the cost of female multiple mating (*ω^2^_f_*) on polyandry evolution when such evolution is driven by sperm limitation caused by sperm competition in the absence of inbreeding depression (Bocedi and Reid 2016). Following from these previous analyses, in the current model we set *ω^2^_f_* = 1.28×10^5^. This represents an intermediate and non-trivial cost of increasing female number of matings for which, in the absence of inbreeding depression in sperm traits, limited polyandry evolves (Fig. 2A, *S* = 0, and Fig. 1B in Bocedi and Reid 2016). For example, given this cost, a female with a re-mating interval phenotype *τ* = 0.5 (i.e. mating with two males per egg), has a probability of dying before the end of the reproductive season of ~ 0.001. For a female with *τ* = 0.2 (i.e. mating with five males per egg), this probability is ~ 0.01. While these costs might seem small, in the absence of mechanisms such as sperm limitation determined by sperm competition and/or inbreeding, they are sufficient to prevent polyandry from evolving (Bocedi and Reid 2016).

*Cost of sperm*

In our current analyses we assume an intermediate cost of sperm (*β* = 0.002), and assume *ω^2^_m_* = 1. This cost of sperm is sufficient to promote evolution of sperm traits that create female sperm limitation, but not sufficiently strong to make female sperm limitation impossible to overcome through evolution of sperm traits (Bocedi and Reid 2016), and it is thus appropriate to investigate the effect of inbreeding depression in sperm traits on polyandry evolution. Here, we explore the sensitivity of our current results to the cost of sperm (*β*)*.* The evolutionary changes in polyandry and sperm traits depended on how costly sperm were to males, relative to the level of female sperm limitation. We explored three scenarios in which sperm can be considered “cheap”, “moderately expensive” and “expensive” relative to the amount of viable sperm required for egg fertilization (*β* = 0.001, 0.002 and 0.003; Bocedi and Reid 2016). In the absence of inbreeding depression (*S* = 0), polyandry only evolved when sperm were moderately costly or costly (Fig. S2.1A). This is because when sperm were cheap, individual males could ensure fertilization to monandrous females (Fig. S2.1B). We previously showed (Bocedi and Reid 2016) that when sperm is moderately expensive (*β* = 0.002), polyandry evolves because of heterogeneity in the males’ strategy, where some males do not provide enough sperm to monandrous females but do not experience survival costs. In the presence of inbreeding depression in sperm number, higher polyandry evolved for a given cost of sperm. Importantly, at moderate to strong inbreeding depression, polyandry evolved also when sperm was cheap relative to sperm limitation (Fig. S2.1A, black lines). These differences in results among different sperm costs are due to decreasing scope for evolutionary compensation in sperm number under increasing cost of sperm. With high cost of sperm, for a given cost of reduced male viability, the same increase in the genotypic value of sperm number corresponds to a much higher increase in sperm mortality rate, and consequently to a lower fertilization probability. Since varying *β* effectively corresponds to varying the level of sperm limitation, it is redundant to also run simulations where *r* is varied.


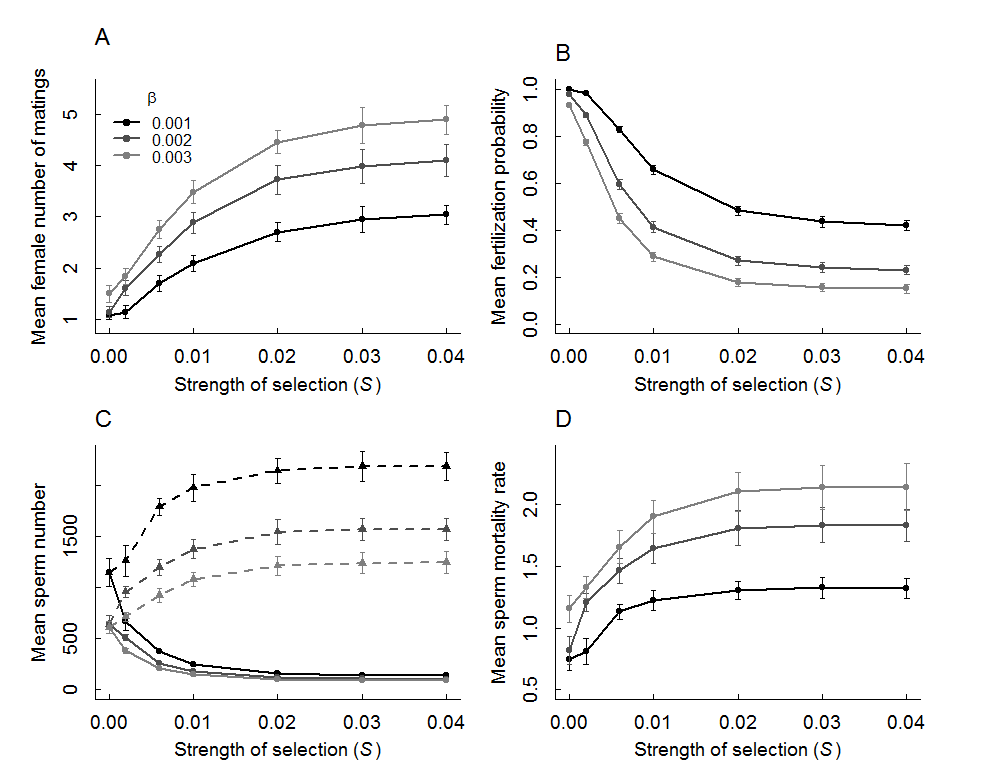


**Figure S2.1.** Greater polyandry evolved when sperm was more costly. (A) Number of female matings per fertilization event (1/*τ*), (B) fertilization probability for a monandrous female (Φ), (C) phenotypic (*s*; circle and solid lines) and genotypic (*g_s_*; triangles and dashed lines) sperm number, and (D) phenotypic sperm mortality rate (*µ*) that evolved at seven strengths of selection against deleterious mutations (*S* = 0, 0.002, 0.006, 0.01, 0.02, 0.03, 0.04) and three costs of sperm (*β* = 0.001, black; 0.002, dark grey; 0.003, light grey). Data show the mean values across all individuals at generation 10,000 averaged across 50 replicates. Bars indicate twice the standard deviation around the replicate means. In all cases, dispersal probability *d* = 0.001.

# S3. Polyandry evolution in the absence of a trade-off between sperm number and sperm mortality rate

To verify that our results are not contingent on the assumption of a trade-off among multiple sperm traits (and specifically between sperm number, *s*, and sperm mortality rate, *µ*), we implemented a model that did not include *µ* and thus considered only one evolving male trait, *s*. Males therefore invest all their reproductive resources *ρ* in *s*, where *ρ_i_* for male *i* is simply given by $\text{ρ}_{\text{i}}\text{ = }\text{β}\text{s}_{\text{i}}$. We retained the cost of sperm determined by *β* (cost of a single sperm cell) and *ω^2^_m_* as in equation 7. Males therefore have to partition their resources only between *s* and their own survival, which is equivalent to simply assuming that sperm is costly. Without *µ* in the model, all sperm cells remain viable from mating to fertilization and males do not have to invest in producing long-living sperm cells. This effectively decreases the overall cost of sperm. However, because without *µ* sperm cells never die and are always viable, we assume that females deplete their sperm reserves completely at the end of each egg’s fertilization, thus avoiding increasing accumulation of sperm across sequential fertilization events.

As in our main model, quantitative results will depend on the cost of sperm (*β*) relative to the level of sperm limitation (*r*). We therefore ran simulations for different levels of sperm limitation (*r* = 0.015 ~ 300 sperm cells for Φ = 0.99; *r* = 0.009 ~ 500 sperm cells; *r* = 0.005 ~ 1000 sperm cells) and for two different costs of sperm (*β* = 0.002 and 0.003).

The qualitative results were consistent whether we included (main model) or excluded the trade-off between *s* and *µ* (compare Fig. 2 with Fig. S3.1, S3.2). Polyandry increased with increasing inbreeding depression in *s* as a consequence of reduced mean fertilization probability (Fig. S3.1A,B and S3.2A,B). In the absence of *µ* we do not observe an evolutionary compensation, in form of increased sperm number genotypic value, for decreased sperm number phenotype due to inbreeding depression (Fig. S3.1C, S3.2C). However, the evolved *s*, even in the absence of inbreeding depression, was higher than in the main model. As expected, greater polyandry evolved in cases of lower egg fertilization probability by a single sperm cell (*r*; Fig. S3.1A and 3.2A, light grey lines) and higher sperm cost (*β*).


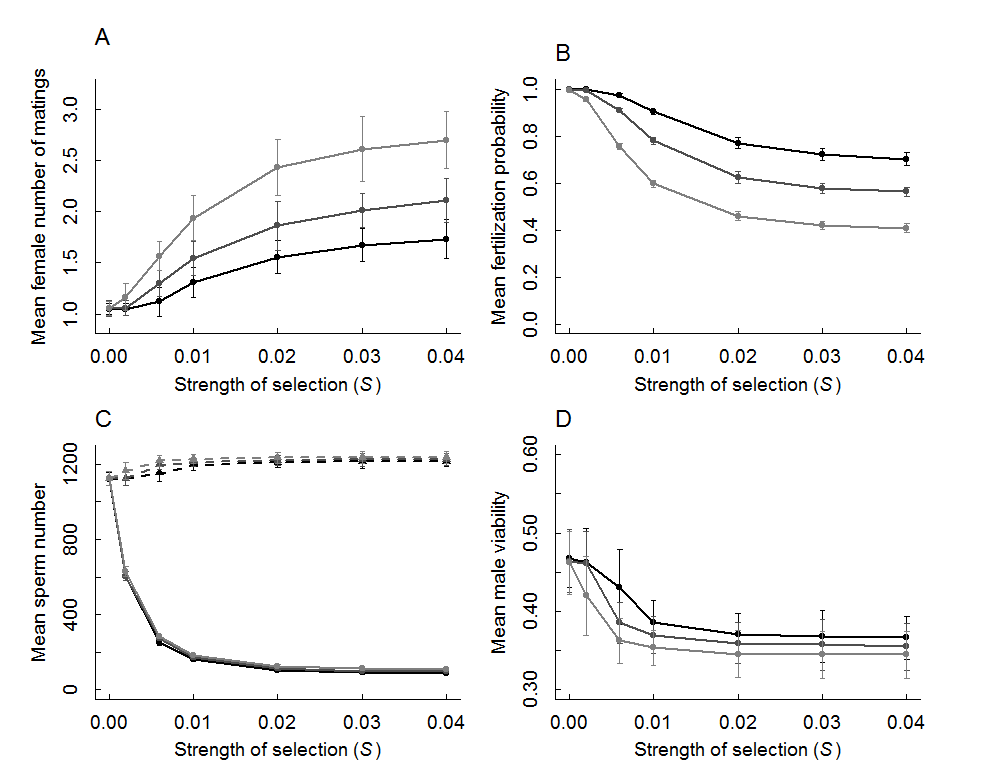


**Figure S3.1.** Polyandry evolved with increasing inbreeding depression in sperm number, and decreasing egg fertilization probability by a single sperm cell (*r* = 0.015, black; 0.009, dark grey; 0.005, light grey). (A) Number of female matings per fertilization event (1/*τ*), (B) fertilization probability for a monandrous female (Φ), (C) phenotypic (*s*; solid lines and circles) and genotypic (*g_s_*; dashed lines and triangles) sperm number and (D) mean male viability at seven strengths of selection against deleterious mutations (*S* = 0, 0.002, 0.006, 0.01, 0.02, 0.03, 0.04), *d* = 0.001 and *β* = 0.002. Data show the mean values across all individuals at generation 10,000 averaged across 50 replicates. Bars indicate twice the standard deviation around the replicate means.


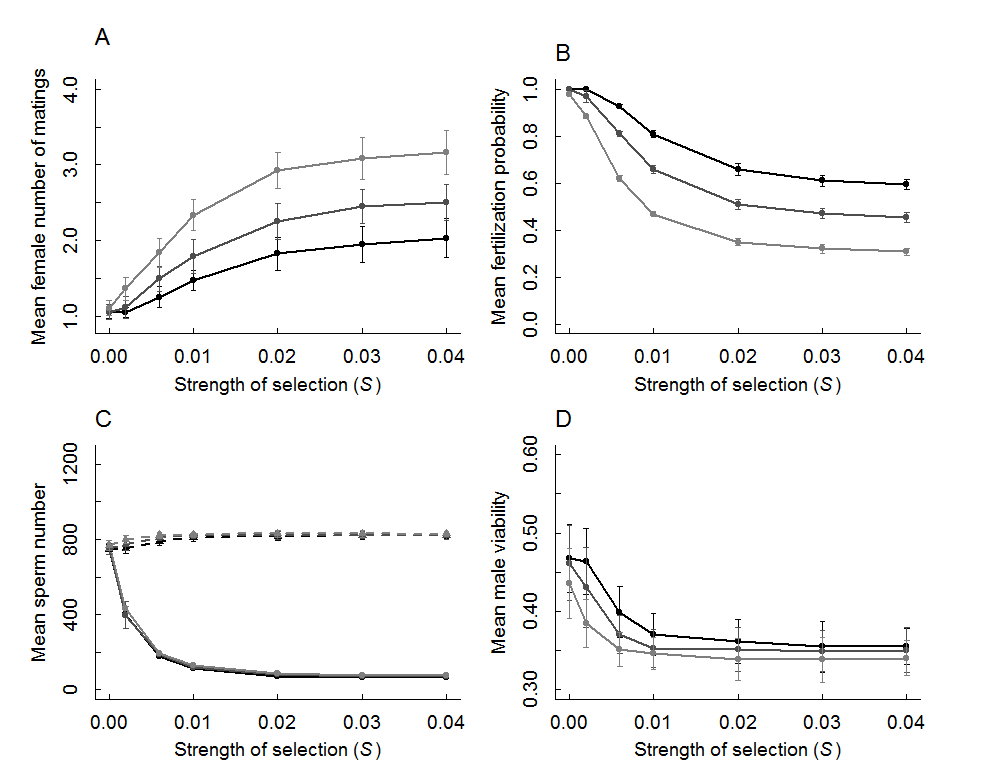


**Figure S3.2.** Greater polyandry evolved given high cost of sperm (*β* = 0.003; cf. Fig. S3.1). (A) Number of female matings per fertilization event (1/*τ*), (B) fertilization probability for a monandrous female (Φ), (C) phenotypic (*s*; solid lines and circles) and genotypic (*g_s_*; dashed lines and triangles) sperm number and (D) mean male viability at seven strengths of selection against deleterious mutations (*S* = 0, 0.002, 0.006, 0.01, 0.02, 0.03, 0.04), three levels of egg fertilization probability by a single sperm cell (*r* = 0.015, black; 0.009, dark grey; 0.005, light grey), and *d* = 0.001. Data show the mean values across all individuals at generation 10,000 averaged across 50 replicates. Bars indicate twice the standard deviation around the replicate means.

# S4. Determinants of female fertility in simulations with and without sperm competition

Female fertility (i.e. mean number of offspring produced per female) was greatly reduced in simulations where we excluded sperm competition (i.e. females that mated multiply mated repeatedly with the same male, Fig. 3F). This was due to two mechanisms. First, because of inbreeding depression in sperm number and males’ reduced investment in sperm relative to simulations with sperm competition, some eggs remained unfertilized (Fig. S4.1A). Second, because they evolved high polyandry to ensure fertility (Fig. 3A), females paid a cost of higher mortality, and hence produced less than *R* = 8 eggs on average (Fig. S4.1B).


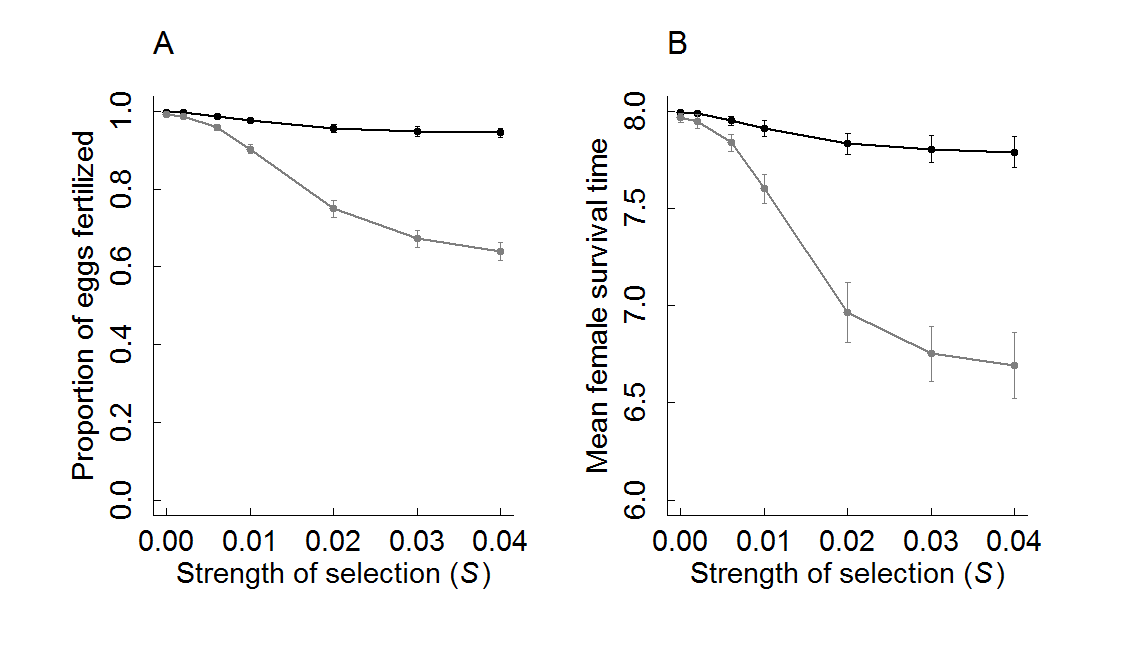


**Figure S4.1.** When female multiple mating evolved in the absence of sperm competition more eggs remained unfertilized and females survived through fewer fertilization events. (A) Mean proportion of a female’s eggs that are fertilized and, (B) the mean number of fertilization events through which females survived, from simulations with (black) and without (grey) sperm competition, at seven strengths of selection against deleterious mutations (*S* = 0, 0.002, 0.006, 0.01, 0.02, 0.03, 0.04) and given dispersal probability *d* = 0.001. Note that because we assume that the total duration of the reproductive phase equals female fecundity *R* (i.e. females lay one egg at a time interval of 1), the mean survival time in (B) corresponds to the mean number of eggs produced by females. Data show the mean values across all females at generation 10,000, averaged across 50 replicates. Bars indicate twice the standard deviation around the replicate means.

# S5. Among-male variance in sperm trait phenotypes.

Increasing strength of selection against deleterious mutations (*S*) strongly reduced the variance in sperm number phenotype both given evolving polyandry (Fig. S5.1A, solid lines) and given fixed monandry (Fig. S5.1A, dashed lines). This explains the reduction in variance in male reproductive success for *S* ≤ 0.006 (*V_m_*, Fig. 4C): despite a slight increase in variance in sperm mortality rate (Fig. S5.1B), males became more similar in their competitive (and absolute) fertilization ability leading to more equally shared paternity among males.

Given evolving polyandry, the variance in sperm number phenotype increased again with further increases in strength of selection (*S* ≥ 0.01; Fig. S5.1A, solid lines). This was due to increasing sperm competition caused by greater evolved polyandry. The increase variance in sperm number phenotype caused the observed increase in *V_m_* (Fig. 4C, solid lines). In contrast, given fixed monandry and hence absence of sperm competition, variance in sperm number phenotype remained very low (Fig. S5.1A, dashed lines), corresponding to reduction or no changes in *V_m_* (Fig. 4C, dashed lines).


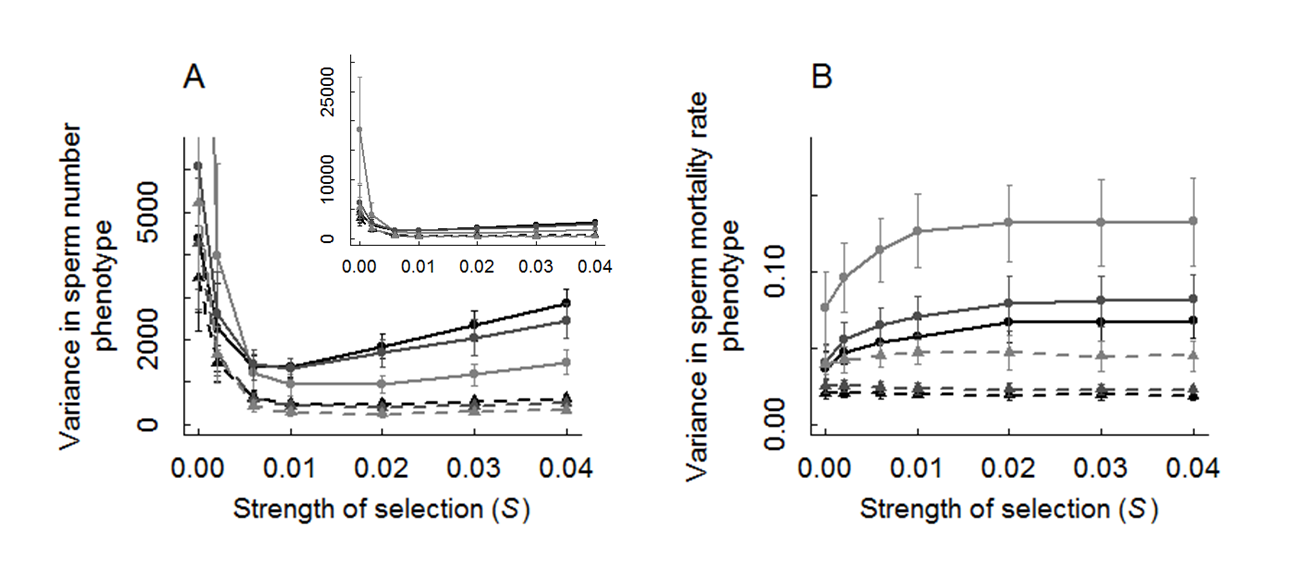


**Figure S5.1.** Variance in (A) sperm number and (B) sperm mortality rate phenotypes in simulations with evolving polyandry (circles and solid lines) versus simulations with fixed monandry (triangles and dashed lines), at seven strengths of selection against deleterious mutations (*S* = 0, 0.002, 0.006, 0.01, 0.02, 0.03, 0.04) and three dispersal probabilities (*d* = 0.1, black; 0.01, dark grey; 0.001, light grey). (A) Without inbreeding depression (*S* = 0) variance in sperm number within replicates, and variance in mean sperm number among replicates, is much larger than with *S* > 0 (inset). The *y*-axis scale in the main panel is adjusted to better visualise the results at *S* > 0. In all panels, data show the mean values at generation 10,000 across 50 replicates. Bars indicate twice the standard deviation around the replicate means.

# S6. Fertilization probability for monandrous females under evolving polyandry versus fixed monandry

Increasing inbreeding depression in sperm number, due to increasing strength of selection against deleterious mutations (*S*) and decreasing dispersal probability (*d*) reduced the mean fertilization probability for a monandrous female (Fig. S6.1). Greater reduction in mean fertilization probability occurred given evolving polyandry (Fig. S6.1, solid lines) compared to fixed monandry (Fig. S6.1, dashed lines).


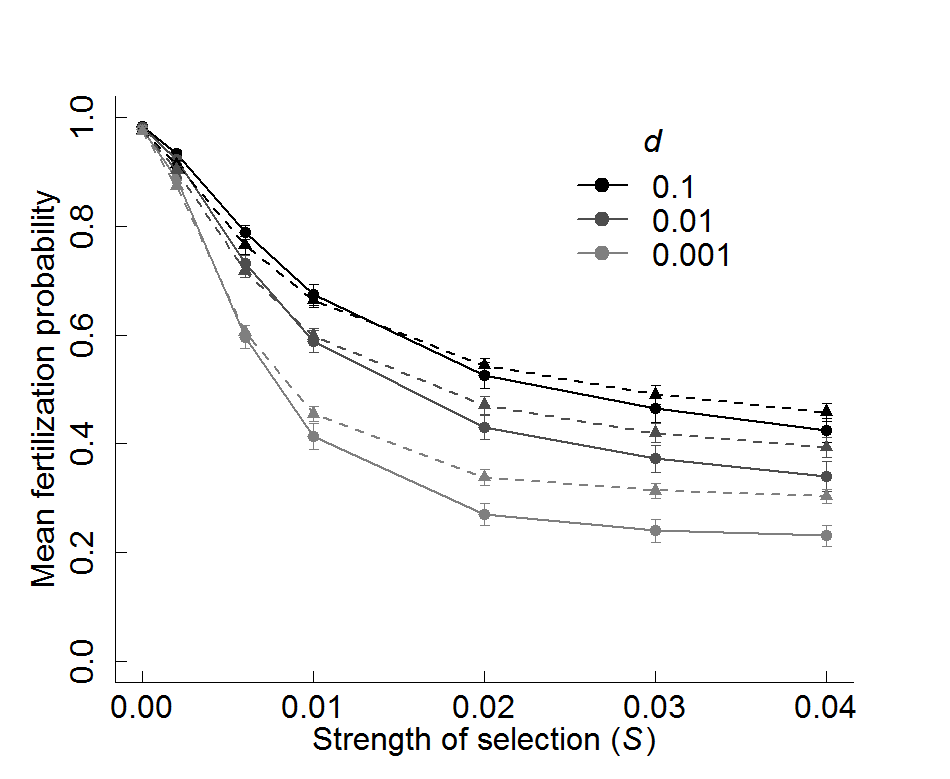


**Figure S6.1.** Mean fertilization probability for a monandrous female (Φ) given evolving polyandry (circles and solid lines) versus fixed monandry (triangles and dashed lines), at seven strengths of selection against deleterious mutations (*S* = 0, 0.002, 0.006, 0.01, 0.02, 0.03, 0.04) and three dispersal probabilities (*d* = 0.1, black; 0.01, dark grey; 0.001, light grey). Data show the mean values across all individuals at generation 10,000, averaged across 50 replicates. Bars indicate twice the standard deviation around the replicate means.

# S7. Inbreeding depression in different male traits.

To test whether emerging evolutionary dynamics of polyandry and sperm traits depend on whether inbreeding depression directly affects specific male sperm traits, or affects males’ overall allocation of costly resources to such traits (Fig. 1B), we ran two additional sets of simulations where inbreeding depression affected sperm mortality rate (*µ*) or the “cost-free” resources (*ρ_0_*) available for allocation to sperm traits instead of sperm number (*s*).

In these simulations, deleterious mutations either increased *µ*, such that:

$\mu_{i}=\frac{g_{\mu,i}}{\left(1-S\right)^{\text{Θ}}\left(1-hS\right)^{\text{θ}}}$, (eqn. S1)

or decreased *ρ_0_*, such that:

$\rho_{0,i}=\rho_{0}\left(1-S\right)^{\text{Θ}}\left(1-hS\right)^{\text{θ}}$. (eqn. S2)

Here, as in equation 1, *S* is the strength of selection against deleterious mutations when homozygous, and Θ and θ are the number of loci that are homozygous and heterozygous for deleterious mutations respectively.

The effect of inbreeding depression on polyandry evolution, and hence on male traits, population-wide homozygosity (H_p_) and female fertility, was similar when inbreeding depression increased *µ* rather than decreased *s* (Fig. S7.1, S7.2). However, when inbreeding depression reduced *ρ_0_*, the increase in polyandry with increasing inbreeding depression was much smaller (Fig. S7.1A). Phenotypic values of *s* and *µ* were respectively higher and lower (Fig. S7.1B,C), determining higher fertilization probability for monandrous females compared to when inbreeding depression affected either sperm trait (Fig. S7.1D), and higher female fertility (Fig. S7.2). However, male viability was much lower when inbreeding depression affected *ρ_0_* (Fig. S7.1E), and the decrease in H_p_ was smaller (Fig. S7.1F). As inbreeding depression affected male fertilization ability only slightly and less polyandry evolved, the effective population size calculated from the sex-specific variances in reproductive success (*N_ev_*) did not change with increasing inbreeding depression, and there was no correlation between a male’s homozygosity and its reproductive success (Fig. S7.3), and thus virtually no change in H_p_.

These effects resulted from differences in how inbreeding depression in sperm traits versus resources affected the initial evolutionary dynamics of male reproductive success, and hence of polyandry. Inbreeding depression in *s* or *µ* caused sudden reductions in male fertilization efficiency (Fig. S7.4D), causing polyandry to evolve to compensate for female sperm limitation (Fig. S7.4A). Resulting sperm competition then drove compensatory evolution of *s* and *µ* (Fig. S7.4B,C, triangles). Conversely, inbreeding depression in *ρ_0_* initially decreased inbred males’ viability, and hence survival, but not their fertilization efficiency (Fig. S7.4D,E). Hence, females did not need to be polyandrous to ensure fertility (Fig. S7.4A), while males had to provide enough sperm to fertilize monandrous females at a cost to their own survival. With strong inbreeding depression in *ρ_0_*, the survival cost meant that males reduced investment into sperm, causing female sperm limitation and consequent polyandry (Fig. S7.1, S7.4).


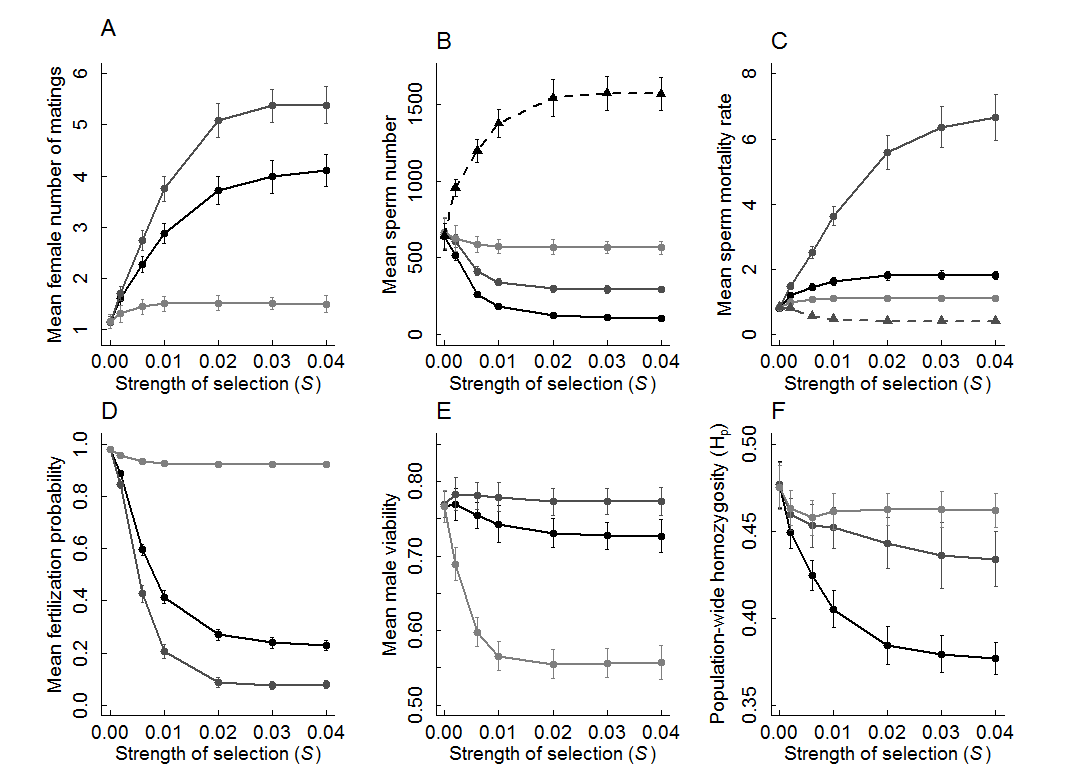


**Figure S7.1.** Higher polyandry evolved when inbreeding depression affected sperm number (*s*, black) or sperm mortality rate (*µ*, dark grey) than when it affected resources available for allocation to sperm (*ρ_0_*, light grey). (A) Number of female matings per fertilization event (1/*τ*), (B) phenotypic (*s*; solid lines and circles) and genotypic (*g_s_*; dashed line and triangles) sperm number, (C) phenotypic (*µ*; solid lines and circles) and genotypic (*g_µ_*; dashed line and triangles) sperm mortality rate, (D) fertilization probability Φ for a monandrous female, (E) male viability and (F) population-wide homozygosity (H_p_), at seven strengths of selection against deleterious mutations (*S* = 0, 0.002, 0.006, 0.01, 0.02, 0.03, 0.04) given dispersal probability *d* = 0.001. Data show the mean values across all individuals at generation 10,000, averaged across 50 replicates. Bars indicate twice the standard deviation around the replicate means.

**
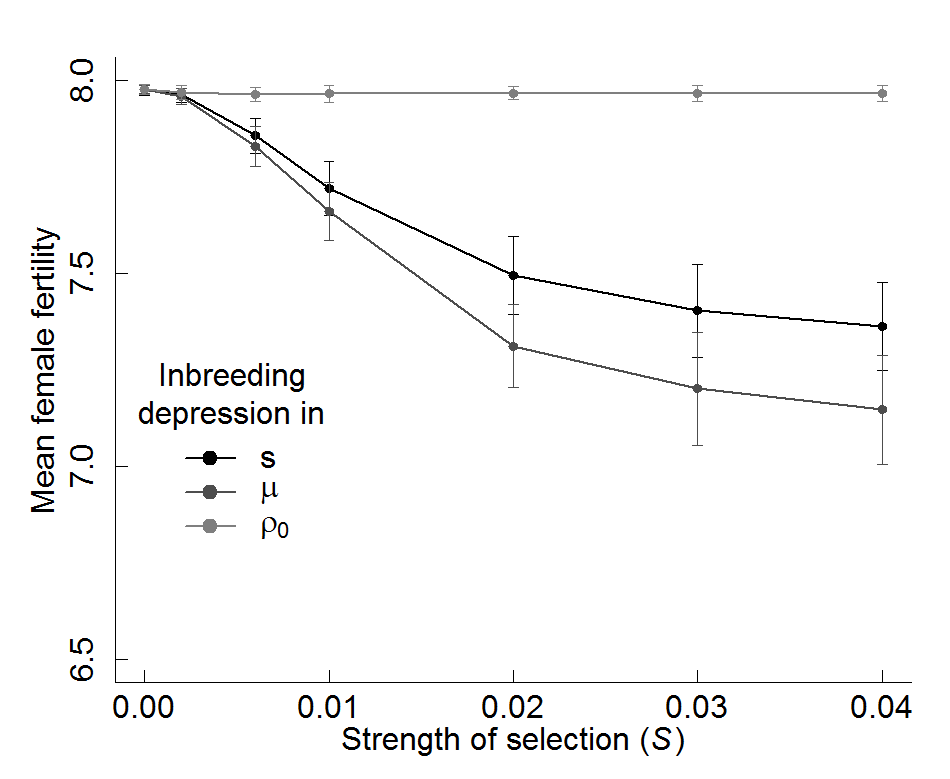
**

**Figure S7.2.** Effect of inbreeding depression in sperm number (*s*, black), sperm mortality rate (*µ*, dark grey) and resources available to males for allocation to sperm (*ρ_0_*, light grey), on female reproductive success. Mean female fertility (i.e. mean female’s number of offspring), at seven strengths of selection against deleterious mutations (*S* = 0, 0.002, 0.006, 0.01, 0.02, 0.03, 0.04,), and dispersal probability *d* = 0.001. Data show the mean values across all individuals at generation 10,000, averaged across 50 replicates. Bars indicate twice the standard deviation around the replicate means.


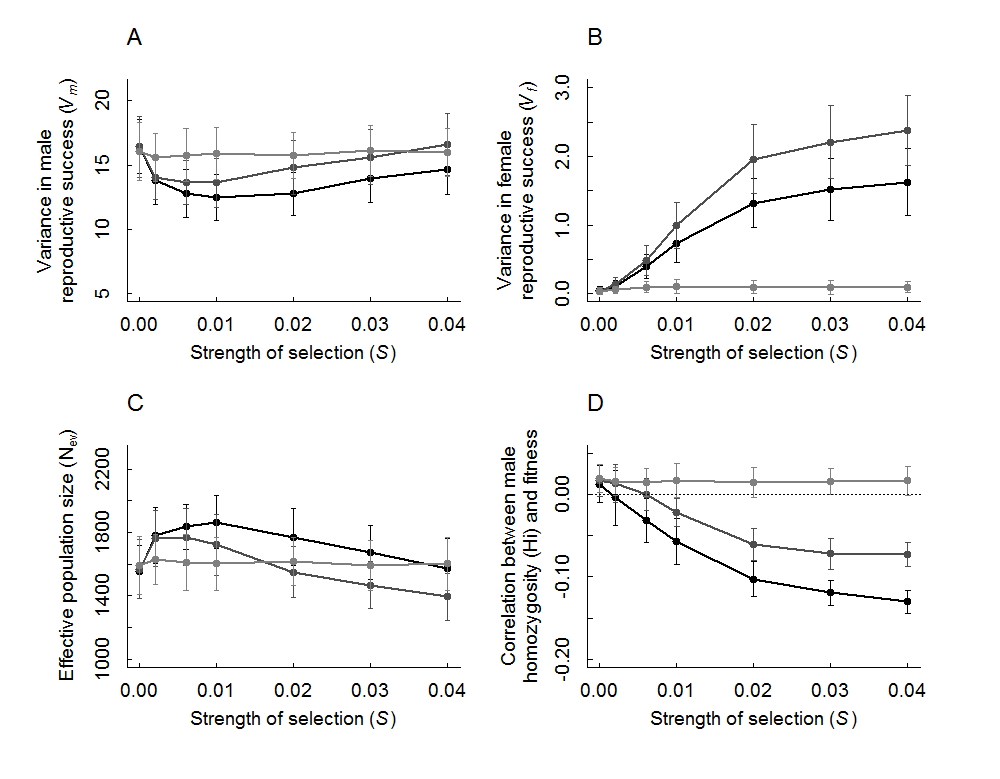


**Figure S7.3.** Variance in (A) male reproductive success (*V_m_*) and (B) female reproductive success (*V_f_*), (C) effective population size (*N_ev_*), and (D) correlation between a male neutral homozygosity (H_i_) and his fitness (number of sired offspring), in simulations with inbreeding depression in sperm number (*s*, black), sperm mortality rate (*µ*, dark grey) and resources available to males for allocation to sperm (*ρ_0_*, light grey), at dispersal probability *d* = 0.001. In (D) Correlations are calculated across males in each sub-population across generations, and then averaged across sub-populations and 50 replicate simulations. The dotted line demarcates zero correlation. In (A-C), data show the mean values at generation 10,000 across 50 replicate simulations. In all panels, bars indicate twice the standard deviation around the replicate means.


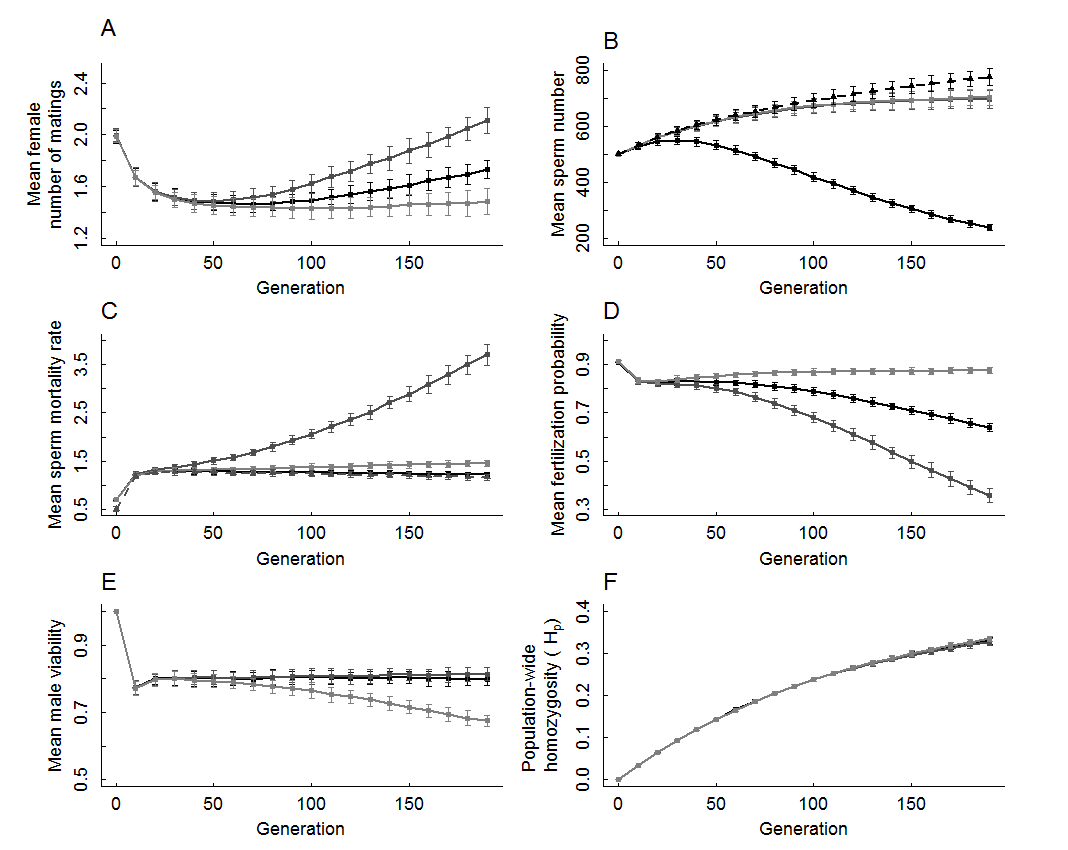


**Figure S7.4.** Effect of inbreeding depression in sperm number (*s*, black), sperm mortality rate (*µ*, dark grey) and resources available to males for allocation to sperm (*ρ_0_*, light grey), on the evolution of polyandry and sperm traits. (A) Number of female matings per fertilization event (1/*τ*), (B) phenotypic (*s*; solid lines and circles) and genotypic (*g_s_*; dashed lines and triangles) sperm number, (C) phenotypic (*µ*; solid lines and circles) and genotypic (*g_µ_*; dashed lines and triangles) sperm mortality rate, (D) fertilization probability for a monandrous female, (E) male viability and (F) population-wide homozygosity (H_p_), given strength of selection *S* = 0.03 and dispersal probability *d* = 0.001. Data show the mean values across all individuals within the first 200 generations (at 10 generation intervals), averaged across 50 replicates. Bars indicate twice the standard deviation around the replicate means.

# References

Bocedi, G., and J. M. Reid. 2016. Coevolutionary feedbacks between female mating interval and male allocation to competing sperm traits can drive evolution of costly polyandry. Am. Nat. 187:334–350.

Charlesworth, D., and J. H. Willis. 2009. The genetics of inbreeding depression. Nat. Rev. Genet. 10:783–96.

Crnokrak, P., and S. C. H. Barrett. 2002. Perspective: purging the genetic load: a review of the experimental evidence. Evolution. 56:2347–2358.

Duthie, A. B., and J. M. Reid. 2016. Evolution of inbreeding avoidance and inbreeding preference through mate choice among interacting relatives. Am. Nat. 188:651–667.

Hedrick, P. W. 1994. Purging inbreeding depression and the probability of extinction: full-sib mating. Heredity. 73:363–372.

Lande, R., D. W. Schemske, and S. T. Schultz. 1994. High inbreeding depression, selective interference among loci, and the threshold selfing rate for purging recessive lethal mutations. Evolution. 48:965–978.

Morton, N. E., J. F. Crow, and H. J. Muller. 1956. An estimate of the mutational damage in man from data on consanguineous marriages. Proc. Natl. Acad. Sci. 42:855–863.

Porcher, E., and R. Lande. 2016. Inbreeding depression under mixed outcrossing, self-fertilization and sib-mating. BMC Evol. Biol. 16:105.

Wang, J., W. G. Hill, D. Charlesworth, and B. Charlesworth. 1999. Dynamics of inbreeding depression due to deleterious mutations in small populations: mutation parameters and inbreeding rate. Genet. Res. 74:165–178.
